# Legionnaires Disease Surveillance in US Department of Veterans Affairs Medical Facilities and Assessment of Health Care Facility Association

**DOI:** 10.1001/jamanetworkopen.2018.0230

**Published:** 2018-06-15

**Authors:** Shantini D. Gamage, Meredith Ambrose, Stephen M. Kralovic, Loretta A. Simbartl, Gary A. Roselle

**Affiliations:** 1 National Infectious Diseases Service, Specialty Care Services, Veterans Health Administration, Department of Veterans Affairs, Washington, DC; 2 Division of Infectious Diseases, Department of Internal Medicine, University of Cincinnati College of Medicine, Cincinnati, Ohio; 3 Cincinnati VA Medical Center, Cincinnati, Ohio

## Abstract

**Importance:**

Legionnaires disease (LD) incidence is increasing in the United States. Health care facilities are a high-risk setting for transmission of *Legionella* bacteria from building water systems to occupants. However, the contribution of LD in health care facilities to national LD rates is not well characterized.

**Objectives:**

To determine the burden of LD in US Department of Veterans Affairs (VA) patients and to assess the amount of LD with VA exposure.

**Design, Setting, and Participants:**

Retrospective cohort study of reported LD data in VA medical facilities in a national VA LD surveillance system from January 1, 2014, to December 31, 2016. The study population included total veteran enrollees and enrollees who used the VA health care system.

**Main Outcomes and Measures:**

The primary outcome was assessment of annual LD rates, categorized by VA and non-VA exposure. Legionnaires disease rates for cases with VA exposure were determined on both population and exposure potential levels. Rates by VA exposure potential were calculated using inpatient bed days of care, long-term care resident days, or outpatient encounters. In addition, types and amounts of LD diagnostic testing were calculated. Case and testing data were analyzed nationally and regionally.

**Results:**

There were 491 LD cases in the case report surveillance system from January 1, 2014, to December 31, 2016. Most cases (447 [91%]) had no VA exposure or only outpatient VA exposure. The remaining 44 cases had VA exposure from overnight stays. Total LD rates from January 1, 2014, to December 31, 2016, increased for all VA enrollees (from 1.5 to 2.0 per 100 000 enrollees; *P* = .04) and for users of VA health care (2.3 to 3.0 per 100 000 enrollees; *P* = .04). The LD rate for the subset who had no VA exposure also increased (0.90 to 1.47 per 100 000 enrollees; *P* < .001). In contrast, the LD rate for patients with VA overnight stay decreased on a population level (5.0 to 2.3 per 100 000 enrollees; *P* < .001) and an exposure level (0.31 to 0.15 per 100 000 enrollees; *P* < .001). Regionally, the eastern United States had the highest LD rates. The urine antigen test was the most used LD diagnostic method; 49 805 tests were performed in 2015-2016 with 335 positive results (0.67%).

**Conclusions and Relevance:**

Data in the VA LD databases showed an increase in overall LD rates over the 3 years, driven by increases in rates of non-VA LD. Inpatient VA-associated LD rates decreased, suggesting that the VA’s LD prevention efforts have contributed to improved patient safety.

## Key Points

**Question:**

What is the Legionnaires disease burden in the US Department of Veterans Affairs medical facilities, a health care system that has prioritized Legionnaires disease prevention with policy?

**Findings:**

In this cohort study, the number of Legionnaires disease cases was low (n = 491) and 91% of cases had no VA exposure or only outpatient VA exposure. Total rates of Legionnaires disease significantly increased from 2014 to 2016, but rates in cases with overnight health care system exposure significantly decreased.

**Meaning:**

Although total Legionnaires disease rates increased, health care system–associated (overnight stay) rates decreased significantly, suggesting that prevention efforts may have contributed to improved patient safety in these settings.

## Teaser

This infection epidemiology study describes the number of reports of Legionnaires disease in VA facilities between 2014 and 2016 and the proportion of cases associated with VA health care facility vs community exposure.

## Introduction

Legionnaires disease (LD) is an acute pneumonia caused by *Legionella* species, primarily *L pneumophila* serogroup 1 in the United States.^1^ The bacteria are naturally present in water,^1^ and infection is associated with exposure from engineered water systems that allow *Legionella* to proliferate. Several prominent outbreaks have increased attention on LD in the United States recently.^2,3,4,5,6^ Legionellosis (including LD and the milder Pontiac fever) is reportable to the Centers for Disease Control and Prevention (CDC); 6079 cases were reported in 2015.^7^ Rates of reported legionellosis have been increasing for decades.^8,9,10^ However, the incidence of LD in the United States and the sources of infection are not well characterized. Underdiagnosis, underreporting, and unknown follow-up of sporadic cases complicate the ability to ascertain a true national picture. The result is an underappreciation for the burden of LD in the United States and missed opportunities for prevention. This is particularly important for health care settings, which have occupants at risk for *Legionella* infection^11^ and for which numerous outbreaks have been described.^2^ The CDC recently reported health care–associated (HCA) LD surveillance data from 21 jurisdictions.^11^ Of the 2809 confirmed LD cases reported in 2015, 3% were classified as definite HCA LD and 17% were classified as possible HCA LD, substantiating health care as a source for exposure. However, national data over multiple years were not reported, categorization of possible HCA LD by inpatient or outpatient exposure was not stated, and data completeness was not known. Furthermore, many hospitals do not perform whole-house surveillance for HCA pneumonia, and guidelines^12,13^ do not specifically indicate *Legionella* diagnostic testing in many patients with HCA pneumonia when it is identified. Therefore, the general burden of HCA LD in the United States may not be well represented by passive surveillance systems.

The Veterans Health Administration (VHA) in the US Department of Veterans Affairs (VA) is the largest integrated health care system in the United States, with more than 1200 sites of care, serving about 6 million veterans annually.^14^ In federal fiscal year (FY) 2016, 91% of veterans using VA benefits were male with a median age of 64 years^15^ and with higher morbidity than in the rest of the United States,^16,17^ which are population factors with higher LD risk.^1,18^ Accordingly, VHA has a *Legionella* prevention policy required at medical facilities to limit *Legionella* growth in water distribution systems and validation of effectiveness using both environmental and clinical surveillance.^19^ The policy promotes LD testing of symptomatic patients and requires heightened awareness for LD when facility water has tested positive for *Legionella*. Concomitant to publication of the policy in 2014, the VHA Central Office implemented a national standardized LD reporting system. This article presents an analysis of data in the system from January 1, 2014, to December 31, 2016, and with no such comparable surveillance database in the United States, provides a unique insight into the rate of LD—both community-associated and HCA—on a national scale.

## Methods

### Setting

Veterans Affairs medical facilities offer a range of services to enrolled veterans across the country and US territories.^20^ During this analysis, VA medical facilities were administratively arranged into 142 health care systems (HCSs), with 170 medical centers^20^ and 132 long-term care sites.^21^

Dissemination of findings that result from review of national VHA operational data sets beyond programmatic needs has been reviewed and approved by the institutional review board at the University of Cincinnati, the institutional review board of record for the Cincinnati VA Medical Center. In this retrospective review of data in an operational surveillance system, there was no greater than minimal risk to patients included in the system, the rights and welfare of patients in the system were not adversely affected, and patient medical records were not altered as a result of this work; these criteria determined that patient consent was not required. This study followed the Strengthening the Reporting of Observational Studies in Epidemiology (STROBE) reporting guideline.

Beginning October 15, 2014, LD data were reported into 2 databases prospectively by personnel at each VA HCS, centrally maintained at the VHA Inpatient Evaluation Center (IPEC).^22^ Legionnaires disease data reporting was required.

#### *Legionella* Case Report Module

The *Legionella* Case Report Module database is used for ad hoc reporting of laboratory-confirmed, community- and VA-associated cases (eAppendix 1 and eTable 1 in the Supplement). Determination of case reporting information provided to this central surveillance system is made at the local facility level. In addition to prospective reporting, retrospective reporting of cases was also required from October 1, 2013, to October 14, 2014.

Legionnaires disease case reports included whether there was exposure to a VA building within the 10 days prior to LD symptom onset. Classification of cases as definitely or possibly associated with VA exposure followed surveillance definitions on the 2014 CDC legionellosis case report form,^23^ with the additional detail of the type of exposure (inpatient, outpatient, or both) if the case was possibly associated with VA. For this article, we assessed VA exposure using the current^23^ and previous^24^ CDC definitions for HCA legionellosis to determine the association of outpatient exposure, a new criterion for the current definitions, with attribution of cases to health care. Definite VA-associated LD cases reported to the database received extensive review and often follow-up with the facility to confirm the diagnosis and classification, and to provide consultative assistance for prevention.

#### *Legionella* Clinical Information Module

The *Legionella* Clinical Information Module database collects monthly reporting of aggregate LD diagnostic information at VA medical facilities, including the number of various LD diagnostic tests performed (eTable 2 in the Supplement). Retrospective reporting of data prior to October 1, 2014, was not required.

### Case Reporting Validation

Data entered into both IPEC databases for each HCS were centrally reviewed monthly to determine if information was complete and consistent across related data elements. Facilities were contacted to update data if necessary. In addition to this routine monthly assessment, system validation was undertaken annually to determine if LD cases were being reported in the *Legionella* Case Report Module as expected (eAppendix 2 in the Supplement).

### Data Analysis

#### Case Surveillance

Cases from January 1, 2014, through December 31, 2016, were reviewed to determine the number of cases reported and the percentage of LD cases associated with the VA. For cases classified as definitely VA associated and possibly VA associated with inpatient stay, the patients’ electronic medical records were reviewed to determine type of exposure (acute care and long-term care) (eAppendix 3 in the Supplement). Of note, cases were assessed for classification as VA associated rather than HCA because we could only reliably ascertain health care contact in the VHA system.

Total LD rates in the VHA patient population for each year were determined using the most recently available VA data on total veteran enrollees in^25^ and enrollees who were users of^25^ the VHA system as population denominators; the first denominator category is comparable with use of the US population by the CDC to determine national rates, while the second denominator category reflects a subset veteran population more likely to have been diagnosed at a VA facility.

Annual rates of community- and VA-associated LD were also calculated. The VA-associated LD rates were assessed separately for patients with overnight stays (inpatient or residential) and patients with only outpatient encounters in the 10 days before symptom onset. For each of these 2 categories, rates were calculated in 2 different ways to account for population-based rates (using unique inpatients or unique outpatients in the patient treatment file data sets and unique residents in the extended care records, as appropriate, for denominator data)^26^ and exposure potential rates (using outpatient encounters in the patient treatment file,^26^ or inpatient bed days of care and long-term care resident days in IPEC,^27,28^ as appropriate, for denominator data). The exposure potential denominators were selected to account for length of stay or amount of contact with health care buildings in a similar manner as is done for other HCA infections.^29^ The numbers of cases, classifications, and rates were also assessed on a regional level for prospectively reported case data (2015 and 2016) using US Census Bureau divisions to delineate US regions^30^ (eAppendix 4 in the Supplement). Denominators for regional rate calculations were derived from state-level VHA data for veteran enrollees who used the VHA system in FY 2015 and FY 2016.^31,32^

#### Diagnostic Testing

Monthly diagnostic tests performed and test results for *Legionella* urine antigen tests (UATs) and clinical cultures were extracted from the IPEC *Legionella* Clinical Information Module for 2015 and 2016. Regional percentage of positivity for each method was calculated using the same US Census Bureau division categories described earlier.

#### Statistical Analysis

National LD rate trends were assessed using Poisson regression analysis. A log-linear regression model was calculated with enrollees, inpatients, outpatients, or bed days of care used as an offset variable. Overdispersion was accounted for using a quasi-likelihood method approach to estimate a dispersion parameter. Pairwise comparisons of regional LD rates were done by χ^2^ analyses (.01 level of significance). The exact results for each pairwise comparison are provided to allow for assessment of the strength of the differences in the presence of multiple comparisons. *Legionella* UAT positivity rates were assessed by χ^2^ analyses for regional and monthly pairwise comparisons. Case fatality rates were compared using the Fisher exact test.

Statistical analyses were performed using SAS version 9.3 (SAS Inc). All analyses were 2-sided and a *P* value of less than .05 was considered significant unless otherwise noted.

## Results

### LD Cases

There were 491 LD cases in the case report surveillance system from January 1, 2014, to December 31, 2016, and the number of cases increased each year (Table 1). Most cases (447 [91%]) had no VA exposure or only outpatient VA exposure in the 10 days prior to symptom onset and the remaining cases (n = 44) had VA exposure with overnight stay. Total LD rates from January 1, 2014, to December 31, 2016, increased for all VA enrollees (from 1.5 to 2.0 per 100 000 enrollees; *P* = .04) and for users of VA health care (2.3 to 3.0 per 100 000 enrollees; *P* = .04). The LD rate for the subset who had no VA exposure also increased (0.90 to 1.47 per 100 000 enrollees; *P* < .001). In contrast, the LD rate for patients with VA overnight stay decreased on a population level (5.0 to 2.3 per 100 000 enrollees; *P* < .001) and an exposure level (0.31 to 0.15 per 100 000 enrollees; *P* < .001). One-third of the cases (163 of 491) had some VA exposure in the 10 days prior to symptom onset. Thirteen of the 491 LD cases (3%) were definitely associated with a VA facility. Most VA-associated cases were in the possible HCA category (150 of 163 [92%]), the majority (119 of 150 [79%]) with only outpatient exposure in the 10 days prior to symptom onset. Definite VA-associated LD cases primarily had exposure in long-term care settings (11 of 13 [85%]) and possible VA-associated cases with overnight stay primarily had acute care exposure (26 of 31 [84%]) (eTable 3 in the Supplement). Five facilities had clusters of LD cases with VA overnight exposure, occurring primarily in 2014 (eTable 4 in the Supplement).

**Table 1.** Distribution of Legionnaires Disease Cases Reported to the Veterans Health Administration Reporting System by VA Association, 2014 to 2016

Case Classification	Cases, No. (%) by Year			
	2014 (n = 136)	2015 (n = 174)	2016 (n = 181)	Total (N = 491)
No VA exposure	82 (60)	113 (65)	133 (73)	328 (67)
Definite VA exposure	7 (5)	5 (3)	1 (1)	13 (3)
Possible VA exposure	47 (35)	56 (32)	47 (26)	150 (31)
Inpatient	5 (4)	5 (3)	2 (1)	12 (2)
Inpatient and outpatient	8 (6)	5 (3)	6 (3)	19 (4)
Outpatient	34 (25)	46 (26)	39 (22)	119 (24)

Abbreviation: VA, Department of Veterans Affairs.

The unadjusted 30-day fatality rate for cases with possible inpatient VA association was higher (8 of 31 [25.8%]; *P* = .004) than the fatality rate for patients with LD with definite VA association (1 of 13 [7.8%]), possible outpatient VA association (8 of 119 [6.7%]), or no VA association (18 of 327 [5.5%]; no death data for 1 patient). Grouped together, the fatality rate for VA patients with overnight stay exposure (definite or possible inpatient) was higher (9 of 44 [20.5%]; *P* = .002) than the fatality rate for patients with outpatient or no VA exposure (26 of 446 [5.5%]; no death data for 1 patient).

Because inclusion of outpatient exposure was new in the 2014 CDC definitions for HCA LD,^23,24^ we examined the impact of the change on attribution of LD cases in the VA data set (Table 2). Including outpatient encounters in the new definition shifted the number of VA-associated cases from 31 to 150, a 384% increase in epidemiologic case attribution. The additional 119 cases classified as possibly VA associated from outpatient-only exposure corresponded with the 27% decrease in classification of cases as community associated.

**Table 2.** Impact of the Change to the CDC Legionellosis Surveillance Definition on the Classification of Cases Reported in the Veterans Health Administration Reporting System From 2014 to 2016

Classification	Previous CDC Definition	Revised CDC Definition	Change in Case Attribution, %
Definitely HCA	Patient hospitalized continuously for ≥10 d before onset of *Legionella* infection	Patient was hospitalized or a resident of a long-term care facility for the entire 10 d prior to onset	
LD cases, No.	13	13	0
Possibly HCA	Patient hospitalized 2-9 d before onset of *Legionella* infection	Patient had exposure to a health care facility for a portion of the 10 d prior to onset	
LD cases, No.	31	150^a^	+384
Community-associated	No inpatient or outpatient hospital visits in the 10 d prior to onset of symptoms	No exposure to a health care facility in the 10 d prior to onset	
LD cases, No.	447	328^b^	−27
Total LD cases, No.	491	491	

Abbreviations: CDC, Centers for Disease Control and Prevention; HCA, health care–associated; LD, Legionnaires disease.

^a^ This increase in the number of possibly HCA LD cases is a result of the inclusion of 119 cases in the Veterans Health Administration reporting system with only outpatient exposure in the 10 days prior to symptom onset. These 119 cases would have been considered community associated using the previous definition. The remaining 31 cases had inpatient-only (n = 12) or inpatient and outpatient (n = 19) exposure and are the same 31 cases counted using the previous definition for possibly HCA.

^b^ This decrease in the number of community-associated LD cases in the Veterans Health Administration reporting system is a result of 119 cases with outpatient-only exposure in the 10 days prior to symptom onset being considered as possibly HCA under the revised definition.

### LD Rates

Total LD rates significantly increased between 2014 and 2016 when calculated using either denominator type (Table 3). Most LD cases were not associated with VA exposure, and the rates significantly increased over the review period (Table 3). In contrast, the rates for the subset of LD cases with inpatient VA exposure significantly decreased when calculated for population level and accounting for exposure potential (Table 3). Outpatient-only VA-associated LD rates were low, and the change over time was not significant regardless of calculation on a population or encounter level (Table 3).

**Table 3.** Legionnaires Disease Cases and Rates in the Veterans Health Administration Health Care System, 2014 to 2016

LD Case Category	Denominator Category	No. of LD Cases/Denominator Value, No.			Rate of LD (per 100 000 Denominator Category)			*P* Value for Poisson Regression
		2014	2015	2016	2014	2015	2016	
Total and non-VA-associated LD cases								
All reported LD cases (non-VA-associated and VA-associated)^a^	Total enrollees in VA HCS	136/9 106 480	174/8 965 923	181/9 046 663	1.5	1.9	2.0	.04
	Enrollees who used the VA HCS in the year	136/5 843 375	174/5 927 103	181/5 995 048	2.3	2.9	3.0	.04
Non-VA-associated LD cases^a^	Total enrollees in VA HCS	82/9 106 480	113/8 965 923	133/9 046 663	0.90	1.26	1.47	<.001
VA-associated LD cases								
LD cases with inpatient and/or LTC VA exposure (includes definite and possible HCA LD)	Unique inpatients and residents per year^b^	20/397 319	15/385 662	9/394 033	5.0	3.9	2.3	<.001
	BDOC (inpatient) and resident days (LTC)^c^	20/6 437 769	15/6 268 381	9/5 998 084	0.31	0.24	0.15	<.001
LD cases with only outpatient VA exposure	Unique outpatients/y^b^	34/5 996 775	46/6 076 638	39/6 142 871	0.57	0.76	0.63	.71
	Outpatient encounters^c^	34/74 214 643	46/76 428 670	39/78 067 260	0.05	0.06	0.05	.78

Abbreviations: BDOC, bed days of care; HCA, health care–associated; HCS, health care system; LD, Legionnaires disease; LTC, long-term care; VA, Department of Veterans Affairs.

^a^ Non-VA-associated cases are patients with LD who did not have contact with a VA health care building in the 10 days prior to symptom onset. It is not possible to reliably know if there was contact with non-VA health care buildings in that period, so this category is not called “community-associated” to avoid potential assumptions of no contact with any health care settings.

^b^ Pertains to population-level calculations.

^c^ Pertains to exposure-level calculations.

Regional differences in LD incidence in the United States have been reported.^9^ We categorized the 355 LD cases reported to the IPEC system in 2015 and 2016 by region using US census divisions and we also found differences (Figure). In all regions, non-VA cases were in the majority. The East North Central (ENC) region reported the most LD cases (including the most definite VA-associated cases) and had the second highest rate (Figure). While the South Atlantic (SA) region had almost the same number of total cases as the ENC region, it had one of the lowest total rates of LD and this rate was significantly lower than the ENC rate (eTable 5 in the Supplement). The Middle Atlantic had the highest rate among the regions, and this rate was significantly higher than 3 other regions (New England, SA, and Pacific) (eTable 5 in the Supplement).

**Figure.**
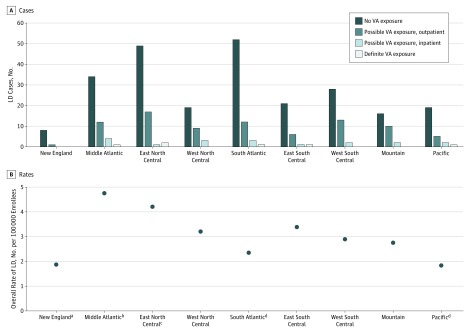
Legionnaires Disease (LD) Cases and Rates for 2015 and 2016, by US Regions A, Numbers of LD cases reported to the Veterans Health Administration tracking system are shown, categorized by type of Veterans Affairs (VA) health care facility exposure, if any. B, Total LD rates were calculated for each region using the number of enrolled veterans in the regions who used the VHA system in the 2-year period as denominator. The results for each pairwise χ^2^ test are provided in eAppendix 4 and eTable 5 in the Supplement to allow for assessment of the strength of the differences in the presence of multiple comparisons. ^a^Rate was significantly different (P < .01) compared with the rate for the Middle Atlantic region by pairwise χ^2^ test. ^b^Rate was significantly different (P < .01) compared with the rates for the New England, South Atlantic, and Pacific regions by pairwise χ^2^ test. ^c^Rate was significantly different (P < .01) compared with the rates for the South Atlantic and Pacific regions by pairwise χ^2^ test. ^d^Rate was significantly different (P < .01) compared with the rates for the Middle Atlantic and East North Central regions by pairwise χ^2^ test.

### Diagnostic Testing

The case reports from 2014 to 2016 indicated that 482 of 491 cases (98%) had a UAT performed, with the majority having a positive result (463 of 482 [96.1%]). For 338 of 491 cases (68.8%), the only *Legionella* diagnostic test performed was the UAT. Clinical culture was done for 109 of 491 cases (22%), and 39 (36%) were positive. Many of the 39 cases with a positive clinical culture result also had a positive UAT result (28 of 39 [72%]); the other cases either had a negative UAT result (6 of 11) or did not have a UAT performed (5 of 11). The few cases without a positive UAT or culture (n = 17) were diagnosed using serology, immunochemistry, or nucleic acid testing.

The Clinical Information Module data set was examined to assess the amount of *Legionella* diagnostic testing performed by VA medical facilities in 2015 and 2016, and these data were categorized by US region and month. In total, 49 805 UATs (Table 4; eTable 6 in the Supplement) and 12 004 clinical *Legionella* cultures were performed nationally in the 2 years with positivity rates of 0.67% (335 positive results) and 0.23% (28 positive results), respectively. All regions had more than 2900 *Legionella* UATs performed except New England (Table 4). Two regions, Middle Atlantic and SA, performed more than 10 000 UATs each (eAppendix 5 in the Supplement), but the UAT positivity rates for these regions were some of the lowest in the country. The highest UAT positivity rate was in the ENC region. See eTable 7 in the Supplement for pairwise χ^2^ analyses of regional UAT positivity. Analysis of UAT data by month showed significantly higher positivity rates in warmer months (Table 4; eTable 8 in the Supplement), despite more UATs performed in cooler months. Clinical culture positivity was low for all regions (eTable 9 in the Supplement).

**Table 4.** *Legionella* Urinary Antigen Testing in Veterans Affairs Medical Facilities in 2015 and 2016^a^

Variable	Positive Tests, No./Tests Performed, No. (%)^b^
US region	
New England	7/1381 (0.51)
Middle Atlantic	41/10 482 (0.39)
East North Central	65/5840 (1.11)
West North Central	32/2991 (1.07)
South Atlantic^c^	70/11 651 (0.60)
East South Central	26/3539 (0.73)
West South Central	43/6034 (0.71)
Mountain	27/4326 (0.62)
Pacific	24/3561 (0.67)
Month	
January	18/5099 (0.35)
February	15/4457 (0.34)
March	20/4876 (0.41)
April	23/4447 (0.52)
May	27/3985 (0.68)
June	32/3545 (0.90)
July	39/3380 (1.15)
August	41/3555 (1.15)
September	43/3714 (1.16)
October	33/3921 (0.84)
November	20/4046 (0.49)
December	24/4780 (0.50)
Total	335/49 805 (0.67)

^a^ See the eAppendix 5 and eTable 6 in the Supplement for a full breakdown of regional testing by month.

^b^ The χ^2^ pairwise comparison results are available for both regional (eTable 7 in the Supplement) and monthly (eTable 8 in the Supplement) data.

^c^ The South Atlantic division includes data from Veterans Health Administration facilities in Puerto Rico. This territory is not included in the US Census Bureau delineation of regions and divisions.^29^

## Discussion

The VHA LD reporting database is unique for collecting information on LD in a nationally distributed US health care system. Reporting indicated that LD was a very infrequent diagnosis regardless of exposure classification. Nonetheless, the overall LD rate in VHA patients significantly increased during the review period, corresponding with the significant increase in the subset of LD cases that had no VA exposure and in alignment with increasing LD rates in the United States.^9,10^ In contrast, the number of LD cases with VA overnight exposure was very low, with significantly decreasing rates over the 3 years. Taken together, these data strongly support that community sources contribute to most of the LD infections in the United States.^10^ The decrease in the LD rate in patients and/or residents with an overnight stay may be a result of intense efforts at VA facilities after publication of the most recent policy in 2014^19^ to prevent *Legionella* growth in building water distributions systems (eAppendix 6 in the Supplement), a supposition similar to a previous review of LD and VHA policy.^33^ While determining a direct correlation between current policy implementation and the decrease in reported cases is beyond the scope of this article, the reduction in case clusters over the 3-year period further suggests improved prevention practices. The results also indicate the particular importance of *Legionella* prevention programs, including LD diagnostic testing, in long-term care settings where high-risk occupants may have prolonged exposure to facility water sources.

The 2015 total LD rate in the VHA enrollee population in this study (1.9 cases per 100 000 persons) was comparable to the 2015 LD rate in the US population for all ages (1.89 cases per 100 000 persons).^7^ To our knowledge, this is the first report of national active surveillance of HCA LD cases in the United States and the first estimation of national HCA LD rates. While there is no known comparator in the US population for HCA LD rates, it is notable that the 2015 rate for VA LD with overnight stay (3.9 cases per 100 000 patients and/or residents) is similar to that in the general population for persons aged 40 to 64 years (2.73 cases per 100 000 persons),^7^ the age range that corresponds with the median age of female (age 46 years) and male (age 64 years) users of VA benefits.^34^ Furthermore, the percentage of LD cases that were definitely VA associated (3%) was similar to recent CDC reporting of definite HCA LD in 21 US jurisdictions.^11^

We did not calculate an overall rate of VA-associated cases because of the variability in the extent and types of exposure to facility water sources between inpatient and/or residential contact and outpatient contact. Furthermore, inclusion of outpatient contact in the possible HCA LD CDC definition is relatively new. This work demonstrates that the definition change, while not affecting the overall number of LD cases, shifted the classification of cases and resulted in an increase in attribution of possible HCA LD with a compensatory decrease in community-associated cases. While perhaps helpful for surveillance, the actual contribution of outpatient and/or transient contact with a health care building to transmission of *Legionella* is uncharacterized.

It has been surmised that the number of LD cases is underdiagnosed in the United States in part because pneumonia cases are often empirically treated with antibiotics. We assessed LD diagnostic testing in the context of *Legionella* prevention policy^19^ that promotes testing beyond guidelines.^12,13^ Increased use of *Legionella* UAT and clinical culture by VA facilities in 2015 and 2016 compared with previously reported testing levels in FY 2012 (15 169 UATs and 3091 clinical cultures)^35^ did not result in the detection of large numbers of LD cases or a marked increase from previously reported numbers of HCA LD in VA.^33,35^ Regions where more than 10 000 UATs were performed in 2015 and 2016 had significantly lower UAT positivity rates in general than other regions that performed less testing. Nonetheless, the data substantiated regional differences in LD rates reported by others^9^ with higher LD rates in general in the eastern part of the United States. While extensive use of UAT did not result in increased UAT positivity rates, the regions that did the most testing in 2015 to 2016 corresponded to regions with the most numbers of LD cases in those same years (compare Table 4 and Figure). The ranking of the regional LD rates in VA are consistent with CDC reporting of regional rates for 2009^9^ for regions with the highest (Middle Atlantic and ENC) and lowest (Pacific) rates. A notable exception is the SA region, which had one of the highest rates in the CDC report for 2009 but one of the lowest rates in the VA.

Increased LD testing occurred in cooler months, perhaps reflecting the higher incidence of respiratory infections in those months. However, as observed by others,^8,9^ the UAT positivity rate for LD was significantly higher in the summer months. Overall, the testing data suggest that heightened awareness for LD in patients with pneumonia may be important on an individual case level for optimal treatment and in regions of the country known to have higher incidence of LD,^36^ but oversensitivity for diagnosis uses resources without necessarily increasing case identification.

### Limitations

This study has limitations. First, facilities may have missed identifying patients for *Legionella* testing. However, because heightened awareness for LD is established by policy and extensive testing is occurring nationally across the system, it is unlikely that a large number of cases were missed. Overall, the VHA requirement for reporting LD cases, the system validation review, and educational outreach provide confidence in the general completeness of the data. Second, patients with LD who were not diagnosed or subsequently cared for at VA medical facilities and had no VA exposure in the 10 days prior to symptom onset are not captured by the reporting system. It is unknown how many cases this represents, and this report focuses on those cases with VA contact and/or care. Third, this work uses facility-reported data from *Legionella* surveillance systems; we did not conduct routine medical record reviews of all reported cases to assess accuracy of the LD diagnosis or to confirm that patients had signs or symptoms of pneumonia. Future work is planned to examine patient demographic characteristics and symptoms as a detailed review of the spectrum of LD. Finally, similar to other surveillance systems, the VHA LD reporting modules did not collect information on implementation of interventions to prevent cases; a comparison of actions at facilities that did and did not have HCA LD cases to determine effective prevention practices was outside the scope of this work.

## Conclusions

This study provides insight into HCA LD in the United States. Perhaps most importantly, these findings from a novel, rigorous reporting program add to the body of knowledge about HCA LD that was previously largely gleaned from local outbreak reports or limited passive surveillance. The number of VA-associated LD cases with overnight exposure was low, with decreasing rates in this category over the study period despite increasing overall rates. This finding sets the stage for additional reviews to assess the contribution of VHA’s proactive *Legionella* prevention policy, including analysis of national environmental *Legionella* data, on the observed improvement in patient safety. Generalization of the results in this study to HCA LD in the United States may be affected by VA population biases. Nonetheless, this study is informative to other health care facilities that are considering implementation of *Legionella* prevention policies. This is particularly relevant because regulatory,^37^ accreditation,^38^ and standards^39^ organizations have recently prioritized water safety in health care.

## Notes

Supplement. **eTable 1.** Data Elements in the VHA Ad Hoc *Legionella* Case Report Database **eTable 2.** VHA Monthly *Legionella* Clinical Information Database Elements for Collection of Aggregate Data on *Legionella* Diagnostic Testing **eTable 3.** Association of Definite Healthcare-Associated Legionnaires Disease (HCA LD) Cases and Possible HCA LD Cases (with inpatient stay) With Acute Care and/or Long Term Care Exposure in the 10 Days Prior to Symptom Onset **eTable 4.** Occurrences of Clusters of LD Cases Reported by VA Medical Facilities in the IPEC *Legionella* Case Report Database **eTable 5.** Pairwise Chi-Square Comparisons of LD Rates Between Regions **eTable 6.** *Legionella* Urinary Antigen Testing in VA Medical Facilities in 2015 and 2016, by Region **eTable 7.** Pairwise Chi-Square Comparisons of *Legionella* Urine Antigen Test Positivity Rates Between Regions **eTable 8.** Pairwise Chi-Square Comparisons of *Legionella* Urine Antigen Test Positivity Rate Between Months **eTable 9.** *Legionella* Clinical Culture in VHA Facilities In 2015 and 2016, by Region **eAppendix 1.** Veterans Health Administration (VHA) Legionnaires Disease (LD) Reporting Databases **eAppendix 2.** Validation That *Legionella* Cases Were Reported by VA Medical Facilities **eAppendix 3.** Additional Epidemiologic Information for Reported Legionnaires Disease (LD) Cases **eAppendix 4.** Analysis of Legionnaires Disease Rates by US Regions **eAppendix 5.** *Legionella* Diagnostic Testing **eAppendix 6.** VHA *Legionella* Prevention Policy
