# Supplementary material for: Legionnaires Disease Surveillance in US Department of Veterans Affairs Medical Facilities and Assessment of Health Care Facility Association
Source: JAMA Netw Open. 2018 Jun 15;1(2):e180230. doi: 10.1001/jamanetworkopen.2018.0230 (PMC6324594; doi:10.1001/jamanetworkopen.2018.0230)
Supplement: Supplement. — eTable 1. Data Elements in the VHA Ad Hoc Legionella Case Report Database eTable 2. VHA Monthly Legionella Clinical Information Database Elements for Collection of Aggregate Data on Legionella Diagnostic Testing eTable 3. Association of Definite Healthcare-Associated Legionnaires Disease (HCA LD) Cases and Possible HCA LD Cases (with inpatient stay) With Acute Care and/or Long Term Care Exposure in the 10 Days Prior to Symptom Onset eTable 4. Occurrences of Clusters of LD Cases Reported by VA Medical Facilities in the IPEC Legionella Case Report Database eTable 5. Pairwise Chi-Square Comparisons of LD Rates Between Regions eTable 6. Legionella Urinary Antigen Testing in VA Medical Facilities in 2015 and 2016, by Region eTable 7. Pairwise Chi-Square Comparisons of Legionella Urine Antigen Test Positivity Rates Between Regions eTable 8. Pairwise Chi-Square Comparisons of Legionella Urine Antigen Test Positivity Rate Between Months eTable 9. Legionella Clinical Culture in VHA Facilities In 2015 and 2016, by Region eAppendix 1. Veterans Health Administration (VHA) Legionnaires Disease (LD) Reporting Databases eAppendix 2. Validation That Legionella Cases Were Reported by VA Medical Facilities eAppendix 3. Additional Epidemiologic Information for Reported Legionnaires Disease (LD) Cases eAppendix 4. Analysis of Legionnaires Disease Rates by US Regions eAppendix 5. Legionella Diagnostic Testing eAppendix 6. VHA Legionella Prevention Policy [file jamanetwopen-1-e180230-s001.pdf]

Supplementary Online Content

Gamage SD, Ambrose M, Kralovic SM, Simbartl LA, Roselle GA. Legionnaires disease surveillance in US Department of Veterans Affairs medical facilities and assessment of health care facility association. *JAMA Netw Open*.
2018;1(1):e180230. doi:10.1001/jamanetworkopen.2018.0230

eTable 1. Data Elements in the VHA Ad Hoc *Legionella* Case Report Database
eTable 2. VHA Monthly *Legionella* Clinical Information Database Elements for Collection of Aggregate Data on *Legionella* Diagnostic Testing
eTable 3. Association of Definite Healthcare-Associated Legionnaires Disease (HCA LD) Cases and Possible HCA LD Cases (with inpatient stay) With Acute Care and/or Long Term Care Exposure in the 10 Days Prior to Symptom Onset
eTable 4. Occurrences of Clusters of LD Cases Reported By VA Medical Facilities in the IPEC *Legionella* Case Report Database
eTable 5. Pairwise Chi-Square Comparisons of LD Rates Between Regions
eTable 6. *Legionella* Urinary Antigen Testing in VA Medical Facilities in 2015 and 2016, by Region
eTable 7. Pairwise Chi-Square Comparisons of *Legionella* Urine Antigen Test Positivity Rates Between Regions
eTable 8. Pairwise Chi-Square Comparisons of *Legionella* Urine Antigen Test Positivity Rate Between Months
eTable 9. *Legionella* Clinical Culture in VHA Facilities In 2015 and 2016, by Region
eAppendix 1. Veterans Health Administration (VHA) Legionnaires Disease (LD) Reporting Databases
eAppendix 2. Validation That *Legionella* Cases Were Reported by VA Medical Facilities
eAppendix 3. Additional Epidemiologic Information for Reported Legionnaires Disease (LD) Cases
eAppendix 4. Analysis of Legionnaires Disease Rates by US Regions
eAppendix 5. *Legionella* Diagnostic Testing
eAppendix 6. VHA *Legionella* Prevention Policy

This supplementary material has been provided by the authors to give readers additional information about their work.

© 2018 Gamage SD et al. *JAMA Network Open*.

eTable 1: Data Elements in the VHA Ad Hoc *Legionella* Case Report Database

Data Element	Reporting Options
**Case Information**	
Station	The reporting station is selected from a drop down menu
Case Number	Automatically assigned by the system when the case is saved
Patient Clue	Patient's first and last initials plus last 4 digits of social security number
**Reporting Information**	
Location	**VA:** case diagnosed at this VHA station<br>**non-VA:** case diagnosed at a non-VA facility
Health Authority	**yes,** case diagnosed at this VHA station and case reported to local public health authority<br>**no,** case diagnosed at this VHA station and case NOT reported to local public health authority<br>**N/A** (case diagnosed at non-VA facility)
**Case Information**	
Symptom onset date	Date of symptom onset of *Legionella* disease [MM-DD-YY]
Diagnosis date	Date of diagnosis of *Legionella* disease [MM-DD-YY]
Pneumonia	**Yes,** patient had radiologic evidence and/or clinical signs/symptoms of pneumonia<br>**No,** patient had NO radiologic evidence and NO clinical signs/symptoms of pneumonia
Death	**Yes,** patient died within 30 days of symptom onset<br>**No,** patient did NOT die within 30 days of symptom onset<br>**Outcome pending,** 30 days has not yet passed
VA Exposure	**Definite VA:** patient had *continuous* contact w/ any VA facility for 10 days prior to symptom onset<br>**Possible VA:** patient had *some* contact w/ any VA facility for 10 days prior to symptom onset<br>**Not VA:** patient had *NO* contact w/ any VA facility in the 10 days prior to symptom onset
Possible Exposure	**Possible-inpt:** inpatient exposure for only a portion of the 10 days prior to symptom onset<br>**Possible-outpt:** outpatient or non-clinical exposure only in the 10 days prior to symptom onset<br>**Possible-both:** both inpt and outpt/non-clinical exposure in the 10 days prior to symptom onset<br>**N/A** (case was not classified as "possible VA")
Total number of VA stations patient was exposed to in the 10 days prior to symptom onset	**1** station<br>**2** stations<br>**3 or more** stations

© 2018 Gamage SD et al. *JAMA Network Open.*

	**N/A** (no VA exposure)
Stations of Exposure	**Reporting:** there was *only* VA exposure at *this* station<br>**Other:** there was *only* VA exposure at *another* station(s)<br>**Both:** there was VA exposure at *both* this station and another station(s)<br>**N/A** (no VA exposure)
**Diagnostic Methodology**	
Urinary Antigen	Positive / Negative / Pending / Not Done
Clinical Culture	Positive / Negative / Pending / Not Done
Serology for *Legionella pneumophila* serogroup 1	Positive / Negative / Pending / Not Done
Serology for non-*Legionella pneumophila* serogroup 1	Positive / Negative / Pending / Not Done
Direct fluorescent antibody (DFA) or Immunohistochemistry (IHC)	Positive / Negative / Pending / Not Done
Nucleic acid assay (e.g. PCR)	Positive / Negative / Pending / Not Done
**Additional Information**	
Building exposure (i.e. whether case was exposed to a VA building that had positive water sample during the most recently completed quarterly environmental water testing)	**Yes,** case was exposed<br>**No,** case NOT exposed

© 2018 Gamage SD et al. *JAMA Network Open*.

eTable 2. VHA Monthly *Legionella* Clinical Information Database Elements for Collection of Aggregate Data on *Legionella* Diagnostic Testing

**Urinary Antigen Testing**	
01	Number of urinary antigen tests performed for the month
02	Number of (01) which were positive
03	Number of patients for whom there were any positive *Legionella* urinary antigen tests
04	Median turnaround time for urinary antigen test results (drop down menu)<br>A – less than 24 hours<br>B – 24 hours to less than 72 hours<br>C – 72 hours to less than 96 hours<br>D – 96 hours or more<br>N/A – not applicable
**Clinical Culture Testing**	
05	Number of *Legionella* clinical cultures performed for the month
06	Number of (05) which were positive
07	Number of patients for whom there were any positive *Legionella* clinical cultures
08	Number of (07) positive for *Legionella spp.* (not further speciated)
09	Number of (07) positive for *Legionella pneumophila* serogroup 1
10	Number of (07) positive for *Legionella pneumophila* serogroup 2-14
11	Number of (07) positive for *Legionella* species not serogrouped
12	Number of (07) positive for *Legionella* species other than *L. pneumophila*
**Serology for *Legionella pneumophila* serogroup 1 (LP-1)**	
13	Number of serology tests for LP-1 performed for the month
14	Number of (13) which were positive
15	Number of patients for whom there were any positive LP-1 serology tests
**Serology for non-*Legionella pneumophila* serogroup 1 (non-LP-1)**	
16	Number of serology tests for non-LP-1 performed for the month
17	Number of (16) which were positive
18	Number of patients for whom there were any positive non-LP-1 serology tests
**Direct Fluorescent Antibody (DFA) or Immunohistochemistry (IHC)**	
19	Number of DFA or IHC tests for *Legionella* performed for the month
20	Number of (19) which were positive
21	Number of patients for whom there were any positive DFA or IHC tests
**Nucleic Acid Assay (e.g. PCR)**	
22	Number of nucleic acid assays (e.g. PCRs) for *Legionella* performed for the month
23	Number of (22) which were positive
24	Number of patients for whom there were any positive nucleic acid assays
***Legionella* Disease Cases**	
25	Number of persons with *Legionella* disease diagnosed during the month
**type of *Legionella* Disease Diagnosis**	
26	Number of (25) with *Legionella pneumophila* serogroup 1
27	Number of (25) with *Legionella pneumophila* serogroup 2-14

© 2018 Gamage SD et al. *JAMA Network Open.*

28	Number of (25) with *Legionella pneumophila* not serogrouped
29	Number of (25) with *Legionella anisa*
30	Number of (25) with *Legionella bozemanii*
31	Number of (25) with *Legionella dumoffii*
32	Number of (25) with *Legionella feeleii*
33	Number of (25) with *Legionella longbeachae*
34	Number of (25) with *Legionella macaecharnii*
35	Number of (25) with *Legionella micdadei*
36	Number of (25) with *Legionella oakridgensis*
37	Number of (25) with other *Legionella* species or unknown
**association to VA Healthcare Facility**	
34	Number of (25) that were classified as non-VA-associated (“community”)
35	Number of (25) that were classified as definitely VA healthcare-associated (HCA)
36	Number of (22) that were classified as possibly VA HCA (inpatient)
37	Number of (25) that were classified as possibly VA HCA (outpatient/non-clinical)
38	Number of (25) that were classified as possibly VA HCA (both inpt and outpt/non-clinical)

© 2018 Gamage SD et al. *JAMA Network Open*.

eTable 3. Association of Definite Healthcare-Associated Legionnaires Disease (HCA LD) Cases and Possible HCA LD Cases (with inpatient stay) With Acute Care and/or Long Term Care Exposure in the 10 Days Prior to Symptom Onset.

	**Location of Patient in 10 days prior to LD symptom onset**											
**HCA LD Case Classification**	**Acute Care**				**Long Term Care**				**Both Acute and Long Term Care**			
	**2014**	**2015**	**2016**	**Total**	**2014**	**2015**	**2016**	**Total**	**2014**	**2015**	**2016**	**Total**
Definite	1	1	0	2	5	3[a]	1	9	1	1	0	2
Possible (Inpatient)	10	7	8	25	2	3	0	5	1	0	0	1
Totals				27				14				3

[a] One patient also had four outpatient appointments in the 10 days prior to symptom onset. HCA LD, Healthcare-associated Legionnaires' disease

© 2018 Gamage SD et al. *JAMA Network Open*.

eTable 4. Occurrences of Clusters[a] of LD Cases[b] Reported By VA Medical Facilities in the IPEC *Legionella* Case Report Database

Facility	Facility Location[c]	Year	No. of Cases	Case Classifications
A	Middle Atlantic	2014	3	One definite VA-associated case<br>Two possible VA-associated cases
B	South Atlantic	2014	3	Three definite VA-associated cases
C	South Atlantic	2014	2	Two possible VA-associated cases
D	East North Central	2014	2	One definite VA-associated case<br>One possible VA-associated case
E	Middle Atlantic	2015	2	Two possible VA-associated cases

[a] Cases are considered to be a cluster if less than 6 months occurs between any two cases.
[b] LD cases with overnight stay at a VA facility were included in this assessment. Cases with only outpatient VA contact or no VA contact were not included.
[c] U.S. census region in which the facility is located.

© 2018 Gamage SD et al. *JAMA Network Open*.

eTable 5. Pairwise Chi-Square Comparisons of LD Rates Between Regions

Overall Chi-square p-value<0.0001									
-	New England	Middle Atlantic	East North Central	West North Central	South Atlantic	East South Central	West South Central	Mountain	Pacific
New England		**0.008**	0.02	0.15	0.51	0.11	0.23	0.32	0.94
Middle Atlantic	**0.008**		0.51	0.08	**0.0001**	0.15	0.02	0.02	**<0.0001**
East North Central	0.02	0.51		0.20	**0.0006**	0.33	0.05	0.05	**0.0001**
West North Central	0.15	0.08	0.20		0.16	0.81	0.68	0.56	0.03
South Atlantic	0.51	**0.0001**	**0.0006**	0.16		0.10	0.29	0.51	0.25
East South Central	0.11	0.15	0.33	0.81	0.10		0.51	0.41	0.02
West South Central	0.23	0.02	0.05	0.68	0.29	0.51		0.81	0.05
Mountain	0.32	0.02	0.05	0.56	0.51	0.41	0.81		0.13
Pacific	0.94	**<0.0001**	**0.0001**	0.03	0.25	0.02	0.05	0.13	

© 2018 Gamage SD et al. *JAMA Network Open*.

eTable 6. *Legionella* Urinary Antigen Testing in VA Medical Facilities in 2015 and 2016, by Region[a]

**Region**	**Jan**	**Feb**	**Mar**	**Apr**	**May**	**Jun**	**Jul**	**Aug**	**Sep**	**Oct**	**Nov**	**Dec**	**Total**
New England													
Tests performed	148	119	130	122	119	99	114	121	98	108	97	106	**1,381**
Tests positive	0	0	0	1	0	1	0	1	1	1	1	1	**7**
% positivity	0.00%	0.00%	0.00%	1.82%	0.00%	2.38%	0.00%	1.43%	0.00%	1.67%	2.08%	1.85%	**0.51%**
Middle Atlantic													
Tests performed	1,077	904	1,037	921	801	736	752	823	810	859	800	962	**10,482**
Tests positive	2	2	0	4	5	3	5	2	10	5	2	1	**41**
% positivity	0.19%	0.22%	0.00%	0.43%	0.62%	0.41%	0.66%	0.24%	1.23%	0.58%	0.25%	0.10%	**0.39%**
East North Central													
Tests performed	584	505	547	541	448	412	400	406	430	497	497	573	**5840**
Tests positive	4	0	3	5	2	4	11	8	11	8	4	5	**65**
% positivity	0.68%	0.00%	0.55%	0.92%	0.45%	0.97%	2.75%	1.97%	2.56%	1.61%	0.80%	0.87%	**1.11%**
West North Central													
Tests performed	297	233	320	296	237	217	187	191	240	237	250	286	**2,991**
Tests positive	0	2	1	0	3	10	4	1	3	2	3	3	**32**
% positivity	0.00%	0.86%	0.31%	0.00%	1.27%	4.61%	2.14%	0.52%	1.25%	0.84%	1.20%	1.05%	**1.07%**
South Atlantic[b]													
Tests performed	1,115	975	1,142	1,012	931	810	807	826	898	957	1,031	1,147	**11,651**
Tests positive	6	4	5	5	5	4	10	7	6	6	5	7	**70**
% positivity	0.54%	0.41%	0.44%	0.49%	0.54%	0.49%	1.24%	0.85%	0.67%	0.63%	0.48%	0.61%	**0.60%**
East South													

© 2018 Gamage SD et al. *JAMA Network Open.*

Central													
Tests performed	336	245	373	320	268	278	235	271	270	247	300	396	**3,539**
Tests positive	1	0	1	2	5	5	3	3	2	2	2	0	**26**
% positivity	0.30%	0.00%	0.27%	0.63%	1.87%	1.80%	1.28%	1.11%	0.74%	0.81%	0.67%	0.00%	**0.73%**
West South Central													
Tests performed	674	636	562	536	538	419	356	396	445	470	446	556	**6,034**
Tests positive	1	1	3	3	5	2	4	9	5	4	1	5	**43**
% positivity	0.15%	0.16%	0.53%	0.56%	0.93%	0.48%	1.12%	2.27%	1.12%	0.85%	0.22%	0.90%	**0.71%**
Mountain													
Tests performed	456	444	404	363	372	317	295	301	315	310	351	398	**4,326**
Tests positive	1	3	3	0	1	3	2	6	2	4	1	1	**27**
% positivity	0.22%	0.68%	0.74%	0.00%	0.27%	0.95%	0.68%	1.99%	0.63%	1.29%	0.28%	0.25%	**0.62%**
Pacific													
Tests performed	412	396	361	336	271	257	234	220	208	236	274	356	**3,561**
Tests positive	3	3	4	3	1	0	0	4	3	1	1	1	**24**
% positivity	0.73%	0.76%	1.11%	0.89%	0.37%	0.00%	0.00%	1.82%	1.44%	0.42%	0.36%	0.28%	**0.67%**
**Total**													
**Tests performed**	**5,099**	**4,457**	**4,876**	**4,447**	**3,985**	**3,545**	**3,380**	**3,555**	**3,714**	**3,921**	**4,046**	**4,780**	**49,805**
**Tests positive**	**18**	**15**	**20**	**23**	**27**	**32**	**39**	**41**	**43**	**33**	**20**	**24**	**335**
**% positivity**	**0.35%**	**0.34%**	**0.41%**	**0.52%**	**0.68%**	**0.90%**	**1.15%**	**1.15%**	**1.16%**	**0.84%**	**0.49%**	**0.50%**	**0.67%**

[a] United States Census Bureau U.S. Regions and Divisions. Located at https://www.census.gov/geo/reference/gtc/gtc_census_divreg.html, last revised February 9, 2015; accessed January 22, 2018.

[b] The South Atlantic division includes data from VHA facilities in Puerto Rico. This territory is not included in the U.S. Census Bureau delineation of Regions and Divisions.

© 2018 Gamage SD et al. *JAMA Network Open*.

eTable 7. Pairwise Chi-Square Comparisons of *Legionella* Urine Antigen Test Positivity Rates Between Regions

	Overall Chi-square *p*-value <0.0001								
	**New England**	**Middle Atlantic**	**East North Central**	**West North Central**	**South Atlantic**	**East South Central**	**West South Central**	**Mountain**	**Pacific**
% positivity	0.51	0.39	1.11	1.07	0.60	0.73	0.71	0.62	0.67
	Pairwise month-by-month comparisons, Chi-square p-values listed below								
	**New England**	**Middle Atlantic**	**East North Central**	**West North Central**	**South Atlantic**	**East South Central**	**West South Central**	**Mountain**	**Pacific**
New England	--	0.52	0.05	0.07	0.64	0.38	0.40	0.62	0.50
Middle Atlantic	0.52	--	**<0.0001**	**<0.0001**	0.02	0.01	**0.005**	0.06	0.03
East North Central	0.05	**<0.0001**	--	0.91	**0.0005**	0.08	0.03	0.01	0.04
West North Central	0.07	**<0.0001**	0.91	--	**0.007**	0.15	0.08	0.04	0.08
South Atlantic	0.64	0.02	**0.0005**	**0.007**	--	0.41	0.42	0.92	0.67
East South Central	0.38	0.01	0.08	0.15	0.41	--	0.90	0.55	0.76
West South Central	0.40	**0.005**	0.03	0.08	0.42	0.90	--	0.59	0.83
Mount	0.62	0.06	0.01	0.04	0.92	0.55	0.59	--	0.78

© 2018 Gamage SD et al. *JAMA Network Open.*

ain									
Pacific	0.50	0.03	0.04	0.08	0.67	0.76	0.83	0.78	--

© 2018 Gamage SD et al. *JAMA Network Open*.

eTable 8. Pairwise Chi-Square Comparisons of *Legionella* Urine Antigen Test Positivity Rate Between Months

Overall Chi-square for monthly difference in test positivity, p<0.0001												
	**Jan**	**Feb**	**Mar**	**Apr**	**May**	**Jun**	**Jul**	**Aug**	**Sep**	**Oct**	**Nov**	**Dec**
**% pos**	0.35	0.34	0.41	0.52	0.68	0.90	1.15	1.15	1.16	0.84	0.49	0.50
Pairwise month-by-month comparisons, Chi-square p-values listed below												
	**Jan**	**Feb**	**Mar**	**Apr**	**May**	**Jun**	**Jul**	**Aug**	**Sep**	**Oct**	**Nov**	**Dec**
**Jan**	-	0.89	0.64	0.22	0.03	**0.0009**	**<0.0001**	**<0.0001**	**<0.0001**	**0.002**	0.30	0.26
**Feb**	0.89	-	0.56	0.19	0.03	**0.001**	**<0.0001**	**<0.0001**	**<0.0001**	**0.002**	0.26	0.22
**Mar**	0.64	0.56	-	0.45	0.08	**0.004**	**<0.0001**	**<0.0001**	**<0.0001**	**0.009**	0.55	0.50
**Apr**	0.22	0.19	0.45	-	0.34	0.04	**0.002**	**0.002**	**0.001**	0.07	0.88	0.92
**May**	0.03	0.03	0.08	0.34	-	0.27	0.03	0.03	0.03	0.40	0.28	0.28
**Jun**	**0.0009**	**0.001**	**0.004**	0.04	0.27	-	0.30	0.30	0.28	0.78	0.03	0.03
**Jul**	**<0.0001**	**<0.0001**	**<0.0001**	**0.002**	0.03	0.30	-	1.00	0.99	0.18	**0.001**	**0.0009**
**Aug**	**<0.0001**	**<0.0001**	**<0.0001**	**0.002**	0.03	0.30	1.00	-	0.99	0.17	**0.001**	**0.0008**
**Sep**	**<0.0001**	**<0.0001**	**<0.0001**	**0.001**	0.03	0.28	0.99	0.99	-	0.16	**0.001**	**0.0007**
**Oct**	**0.002**	**0.002**	**0.009**	0.07	0.40	0.78	0.18	0.17	0.16	-	0.06	0.05
**Nov**	0.30	0.26	0.55	0.88	0.28	0.03	**0.001**	**0.001**	**0.001**	0.06	-	0.96
**Dec**	0.26	0.22	0.50	0.92	0.28	0.03	**0.0009**	**0.0008**	**0.0007**	0.05	0.96	-

LD clinical culture testing

The monthly number of clinical culture tests performed for LD diagnosis and the number that were positive is shown in eTable 9 for 2015 and 2016. Results are categorized by U.S. Census Division. One region (Middle Atlantic) stands out for doing over 5,600 culture tests in that time period. Notably, one healthcare system in that region did a large amount of the testing with 3,189 culture tests performed.

© 2018 Gamage SD et al. *JAMA Network Open.*

eTable 9. *Legionella* Clinical Culture in VHA Facilities in 2015 and 2016, By Region[a]

	**Jan**	**Feb**	**Mar**	**Apr**	**May**	**Jun**	**Jul**	**Aug**	**Sep**	**Oct**	**Nov**	**Dec**	**Total**
New England													
Tests performed	9	0	2	3	0	1	1	4	4	2	0	1	**27**
Tests positive	0	0	0	0	0	0	0	0	0	0	0	0	**0**
% positivity	0.00%	N/A	0.00%	0.00%	N/A	0.00%	0.00%	0.00%	0.00%	0.00%	N/A	0.00%	**0.00%**
Middle Atlantic													
Tests performed	541	470	466	516	529	529	466	498	402	418	353	458	**5,646**
Tests positive	3	1	0	1	1	1	0	0	5	1	0	0	**13**
% positivity	0.55%	0.21%	0.00%	0.19%	0.19%	0.19%	0.00%	0.00%	1.24%	0.24%	0.00%	0.00%	**0.23%**
East North Central													
Tests performed	126	127	148	119	141	102	98	96	115	111	111	135	**1,429**
Tests positive	1	0	0	0	0	0	1	1	0	0	0	0	**3**
% positivity	0.79%	0.00%	0.00%	0.00%	0.00%	0.00%	1.02%	1.04%	0.00%	0.00%	0.00%	0.00%	**0.21%**
West North Central													
Tests performed	11	16	9	15	15	23	15	17	23	18	8	37	**207**
Tests positive	0	0	0	0	0	0	1	0	0	0	0	0	**1**
% positivity	0.00%	0.00%	0.00%	0.00%	0.00%	0.00%	6.67%	0.00%	0.00%	0.00%	0.00%	0.00%	**0.48%**
South Atlantic[b]													
Tests performed	74	79	102	105	80	78	68	47	72	70	71	101	**947**
Tests positive	0	2	0	0	0	0	2	1	0	0	0	0	**5**
% positivity	0.00%	2.53%	0.00%	0.00%	0.00%	0.00%	2.94%	2.13%	0.00%	0.00%	0.00%	0.00%	**0.53%**
East South													

© 2018 Gamage SD et al. *JAMA Network Open.*

Central													
Tests performed	9	5	15	4	3	1	4	6	11	2	5	3	**68**
Tests positive	0	0	0	0	0	0	0	0	0	0	0	0	**0**
% positivity	0.00%	0.00%	0.00%	0.00%	0.00%	0.00%	0.00%	0.00%	0.00%	0.00%	0.00%	0.00%	**0.00%**
West South Central													
Tests performed	55	60	33	31	30	37	26	39	65	73	53	67	**569**
Tests positive	0	0	0	0	0	0	1	0	0	2	0	1	**4**
% positivity	0.00%	0.00%	0.00%	0.00%	0.00%	0.00%	3.85%	0.00%	0.00%	2.74%	0.00%	1.49%	**0.70%**
Mountain													
Tests performed	92	138	137	90	107	83	91	114	107	116	89	141	**1,305**
Tests positive	0	0	0	0	0	0	0	0	0	0	0	0	**0**
% positivity	0.00%	0.00%	0.00%	0.00%	0.00%	0.00%	0.00%	0.00%	0.00%	0.00%	0.00%	0.00%	**0.00%**
Pacific													
Tests performed	212	186	203	197	149	142	134	114	102	106	123	138	**1,806**
Tests positive	0	0	1	0	0	0	0	0	1	0	0	0	**2**
% positivity	0.00%	0.00%	0.49%	0.00%	0.00%	0.00%	0.00%	0.00%	0.98%	0.00%	0.00%	0.00%	**0.11%**
**Total**													
**Tests performed**	1,129	1,081	1,115	1,080	1,054	996	903	935	901	916	813	1,081	**12,004**
**Tests positive**	4	3	1	1	1	1	5	2	6	3	0	1	**28**
**% positivity**	0.35%	0.28%	0.09%	0.09%	0.09%	0.10%	0.55%	0.21%	0.67%	0.33%	0.00%	0.09%	**0.23%**

[a] United States Census Bureau U.S. Regions and Divisions. Located at https://www.census.gov/geo/reference/gtc/gtc_census_divreg.html, last revised February 9, 2015; accessed January 22, 2018.

[b] The South Atlantic division includes data from VHA facilities in Puerto Rico. This territory is not included in the U.S. Census Bureau delineation of Regions and Division.

© 2018 Gamage SD et al. *JAMA Network Open*.

eAppendix 1. Veterans Health Administration (VHA) Legionnaires' Disease (LD) Reporting Databases

VHA Central Office implemented two different reporting databases in October, 2014 to collect LD case and diagnostic information from VA medical facilities in the national VHA healthcare system. The reporting databases are maintained by the VHA Inpatient Evaluation Center (IPEC). The *ad hoc* IPEC *Legionella* case report database collects information on *Legionella* cases (See eTable 1 for database reporting elements). A laboratory-confirmed LD case is reported into the tracking database if the diagnosis 1) occurs at a VA medical facility, 2) occurs at a non-VA facility but the patient had exposure to a VA facility within the 10 days prior to symptom onset, or 3) occurs at a non-VA facility but the patient is cared for in the acute phase of the illness at a VA facility. The IPEC Clinical Information database collects monthly aggregate data on *Legionella* diagnostic testing and results (See eTable 2 for database reporting elements).

© 2018 Gamage SD et al. *JAMA Network Open*.

eAppendix 2. Validation That *Legionella* Cases Were Reported by VA Medical Facilities

Validation Method

The VHA national electronic health record (Patient Treatment Files [PTF] and Extended Care databases) were queried for the International Classification of Diseases, Ninth Revision, Clinical Modification (ICD-9-CM) diagnosis code 482.84 to identify VHA acute care patients and long term care residents with a discharge diagnosis of LD in FY 2014 and FY 2015. For FY 2016 and the first three months of FY 2017 (to cover calendar year 2016), these same health record databases were queried for the ICD-10-CM LD diagnosis code A48.1. The medical charts of patients and residents identified by the ICD-9-CM or ICD-10-CM queries, but who were not reported as cases of LD to the IPEC Case Report database, were reviewed to determine if the cases should have been reported to the database. This review was done independently in duplicate and discordant case reporting determinations were assessed by a third person. For cases deemed as "missing" from the IPEC database, VA healthcare facilities were contacted to review patient charts and enter these cases into the database, as appropriate. Since outpatient administrative coding in general may be less reliable for identifying patients[1], a subset of ICD-9-CM extractions of outpatients with the LD diagnosis code was reviewed for FY 2014 and FY 2015 (10 extractions per year) to determine if further review was necessary.

Validation Results

The validation review of acute care (inpatient) and long term care settings found a lower number of missed cases in the IPEC system in the prospective reporting that started on October 15, 2014 (10 missed cases out of 32 ICD-9-CM extracts reviewed for 2015; 12 missed cases out of 39 ICD-10-CM extracts reviewed for 2016), compared to the retrospective reporting to the system the year prior (40 missed cases out of 57 extracts reviewed for 2014). The majority of the missed cases were not associated with the VA or only had outpatient exposure (10/10 and 12/12 for prospective reporting in 2015 and 2016, respectively; 37/40 for retrospective reporting in 2014). For the subset outpatient review using the ICD-9-CM query, 10 of 126 unique ICD-9-CM extracts were reviewed for FY 2014 and 10 of 81 unique extracts were reviewed for FY 2015. The majority of the ICD-9 extracts (19/20 reviewed) were not considered missed patients (e.g. cases were already identified in the inpatient or long term care data sets, cases had a past diagnosis of LD, or there was no LD diagnosis in the medical chart). Therefore, it was determined that further outpatient review for 2014 and 2015, and any outpatient review in 2016, was not necessary to capture substantial numbers of missed cases.

Validation Summary

The annual system validation process demonstrated the challenges of retrospective (2014) versus prospective (2015 and 2016) collection of case data, the value of secondary review of a new system, and the necessity of follow-up on data entry to ensure accuracy and reinforce implementation rules. Implementation of routine and annual validation processes provides confidence in comprehensive case reporting to the system.

Reference

1. Fang MC, Fan D, Sung H, et al. Validity of using inpatient and outpatient administrative codes to identify acute venous thromboembolism: the CVRN VTE study. *Med. Care* 2017; 55(12):e137-e143.

© 2018 Gamage SD et al. *JAMA Network Open*.

eAppendix 3. Additional Epidemiologic Information for Reported Legionnaires Disease (LD) Cases

Assessment of LD cases by type of VA exposure

The electronic health records of LD patients with overnight stays in the 10 days prior to LD symptom onset were assess for the type of VA exposure (acute care, long term care, or both) during those 10 days (eTable 3).

Clusters of LD Cases at Facilities

Data in the IPEC *Legionella* Case Report database were reviewed for any clusters of LD cases with overnight VA stay (i.e. definite VA-associated cases and possible VA-associated cases with inpatient stay). Cases were included in a cluster if any two occurred within a 6 month period. Five clusters of cases occurred at five different VA medical facilities (eTable 4), with no more than 3 cases within a cluster. Four of the five clusters were reported retrospectively for 2014 (Facilities A, B, C, and D), with most cases occurring prior to, or just after, promulgation of VHA Directive 1061. Since publication of the Directive and subsequent implementation of proactive LD case reporting, centralized consultative assistance is consistently available and provided for LD cases definitely or likely associated with a VA facility. This assistance is provided by electronic mail, teleconference and/or site visit, as determined in conjunction with the local facility and as appropriate for the situation. Of note, only one facility had a cluster of cases that included more than one definite VA-associated case (Facility B).

© 2018 Gamage SD et al. *JAMA Network Open.*

eAppendix 4. Analysis of Legionnaires Disease (LD) Rates by US Regions

Categorization of states into regions

For regional comparisons of LD and diagnostic testing rates, we used U.S. Census divisions (https://www.census.gov/geo/reference/gtc/gtc_census_divreg.html) as the basis to categorize the states and territories into regions as follows: New England: Connecticut, Maine, Massachusetts, New Hampshire, Rhode Island, Vermont; Middle Atlantic: New Jersey, New York, Pennsylvania; South Atlantic: Delaware, DC, Florida, Georgia, Maryland, North Carolina, Puerto Rico, South Carolina, Virginia, West Virginia; East North Central: Illinois, Indiana, Michigan, Ohio, Wisconsin; East South Central: Alabama, Kentucky, Mississippi, Tennessee; West South Central: Arkansas, Louisiana, Oklahoma, Texas; West North Central: Iowa, Kansas, Minnesota, Missouri, Nebraska, North Dakota, South Dakota; Mountain: Arizona, Colorado, Idaho, Montana, Nevada, New Mexico, Utah, Wyoming; Pacific: Alaska, California, Hawaii, Oregon, Washington. Of note, since U.S. territories are not included in the Census Bureau's divisions, data from the VA Caribbean Healthcare System in Puerto Rico were included in the South Atlantic region to best align with the VHA regional category for this territory.

Statistical analysis of LD rates by regions

Since multiple regions are compared by pairwise Chi-square analysis, eTable 5 is provided to show the p-value for each comparison. Rather than adjusting the p-value for multiple comparisons, we feel that providing the exact pairwise results allows the reader to informally choose his/her own level of statistical significance and balance the risks of false positive and false negative results. Values below the 0.01 alpha threshold are bolded in the table.

© 2018 Gamage SD et al. *JAMA Network Open*.

eAppendix 5. *Legionella* Diagnostic Testing

Urine Antigen Testing

Table 4 in the paper shows the regional and monthly UAT total testing volume and positivity rates for 2015-2016. eTable 6 below provides the details of the regional testing by month. Two regions stand out for doing over 10,000 UATs in that time period: the Middle Atlantic and South Atlantic regions. Notably, one healthcare system in each of those regions did a large amount of the testing. In the Middle Atlantic region, one healthcare system did 2,822 UATs. In the South Atlantic Region, one healthcare system did 1527 UATs. eTable 7 and eTable 8 indicate the results of the pairwise Chi-square analyses of urine antigen positivity rates corresponding to the data in Table 4 in the manuscript and eTable 6 below. The reason(s) for the variability in the amount of testing between the regions was not available in the surveillance data collected. One possibility is a potential difference in patient demographics (i.e. at-risk populations) in the various regions. Another possibility is that more testing was done in localities with a known higher incidence of LD. Regardless, regions with higher testing did not necessarily result in detection of more cases, and the percent positivity was low in all regions.

It is noted that UAT testing increased by about 8% from 2015 (n=23,897) to 2016 (n=25,908), and it could be surmised that the increases in non-VA associated LD rates between 2014 and 2016 are a result of increased testing. However, non-VA LD increased by about 18% from 2015 to 2016 (see Table 3 in the paper). Therefore, while some of the increase in cases in later years may be because of increased testing, the larger increase in the number of cases compared to the increase in testing suggests that other factors, such as a true increase in incidence, are involved.

© 2018 Gamage SD et al. *JAMA Network Open.*

eAppendix 6. VHA *Legionella* Prevention Policy

Overview

VHA has had longstanding policy for prevention of Legionnaires' disease at its medical facilities. Past policy focused mainly on implementation of hot water temperatures to limit *Legionella* growth in building water systems while also requiring measures to limit the risk of scald injury. In 2008, a separate *Legionella* policy was promulgated requiring *Legionella* risk assessments at VHA medical facilities.[1] At the time of the LD case reporting in this paper, VA healthcare systems were required to follow the updated policy, VHA Directive 1061, Prevention of healthcare-associated *Legionella* disease and scald injury from potable water distribution systems (published August 13, 2014). This policy was a comprehensive document that combined actions for prevention of *Legionella* growth in building water systems (e.g., water temperature, biocide, improved water flow) with actions to assess and mitigate risk and validate implementation of controls. In essence, VHA Directive 1061 combined elements of the two previous VHA policies; a critical additional requirement was the institution of a water safety committee at each medical facility to oversee program implementation and respond to validation triggers. The principles of VHA Directive 1061 are in alignment with guidance from the World Health Organization[2] and ANSI/ASHRAE Standard 188[3].

VHA Directive 1061, a work of the U.S. federal government, is publicly available for five years after publication at this website: https://www.va.gov/vhapublications/ViewPublication.asp?pub_ID=3033 (accessed January 2, 2018). It is also copied below in its entirety.

References

1. Lin YE, Stout JE, Yu VL. Prevention of hospital-acquired legionellosis. *Curr. Opin. Infect. Dis*. 2011; 24(4):350-356.
2. World Health Organization. Water safety in buildings. Published March, 2011. Available at: http://www.who.int/water_sanitation_health/publications/2011/9789241548106/en/ . Accessed 2/26/2018.
3. ASHRAE. ANSI/ASHRAE Standard 188-2015. Legionellosis: risk management for building water systems. 2015. Available at: https://www.ashrae.org/. Accessed 2/26/2018.

Department of Veterans Affairs **VHA Directive 1061**
Veterans Health Administration **Transmittal Sheet**
Washington, DC 20420 **August 13, 2014**

PREVENTION OF HEALTHCARE-ASSOCIATED *LEGIONELLA* DISEASE AND SCALD INJURY FROM POTABLE WATER DISTRIBUTION SYSTEMS

© 2018 Gamage SD et al. *JAMA Network Open*.

1. REASON FOR ISSUE: This Veterans Health Administration (VHA) Directive addresses the prevention of healthcare-associated *Legionella* Disease and Scald Injury from Potable Water Distribution Systems in VHA buildings.
2. SUMMARY OF CONTENTS: This Directive establishes policy for the prevention and control of healthcare-associated *Legionella* disease in VHA-owned buildings in which patients, residents, or visitors stay overnight.
3. RELATED ISSUES: None.
4. RESPONSIBLE OFFICE: The Deputy Under Secretary for Health for Operations and Management (10N) is responsible for the contents of this Directive. Questions related to the engineering aspects of this Directive are to be directed to the Office of Capital Asset Management, Engineering, and Support (10NA5) at 202-632-7900. Questions related to clinical aspects and validation processes in this Directive are to be directed to the National Infectious Diseases Service (10P4E) in the Office of Patient Care Services (10P4) at 513-246-0270.
5. RESCISSIONS: VHA Directive 2008-010, dated February 11, 2008, VHA Directive 2009-009, dated February 25, 2009, and Information Letter 10-2013-006, dated May 3, 2013 are rescinded.
6. RECERTIFICATION: This VHA Directive is scheduled for recertification on or before the last working day of August 2019.
Carolyn M. Clancy, MD
Interim Under Secretary for Health
DISTRIBUTION: E-mailed to the VHA Publications Distribution List on 08/15/2014.

PREVENTION OF HEALTHCARE-ASSOCIATED *LEGIONELLA* DISEASE AND SCALD INJURY FROM POTABLE WATER DISTRIBUTION SYSTEMS

1. PURPOSE: This Veterans Health Administration (VHA) Directive establishes policy for the prevention and control of healthcare-associated *Legionella* disease in VHA-owned buildings in which patients, residents, or visitors stay overnight. These types of buildings include, but are not limited to, acute care facilities, Community Living Centers (CLCs), domiciliaries, and Fisher Houses and other temporary lodging facilities (e.g. "hoptels").
AUTHORITY: 38 U.S.C. 7301(b).
***NOTE:** This Directive was developed to address areas in healthcare with a recognized higher risk for Legionella disease. It is anticipated that this Directive, and information gathered from its implementation, will serve as a template for further prevention policy in other VHA healthcare settings. Policy and guidance for full case investigations of confirmed or suspected healthcare-associated Legionella disease is not encompassed within the scope of this Directive.*

2. BACKGROUND:
a. *Legionella* is a Gram-negative bacterium, which causes respiratory diseases collectively referred to as legionellosis. Legionellosis includes *Legionella* pneumonia, traditionally known as Legionnaires' disease and hereafter abbreviated as "LD" for "*Legionella* disease", and Pontiac Fever, a self-limiting respiratory illness. Disease is primarily caused by *Legionella pneumophila*; however, other species of *Legionella* can be pathogenic, particularly in transplant and other immunocompromised or high risk

© 2018 Gamage SD et al. *JAMA Network Open*.

patients. The bacteria, found naturally in water, have been associated with disease from building water distribution systems. LD occurs after inhalation or aspiration of contaminated water, followed by a general incubation period of 2 to 14 days. *Legionella* bacteria are not transmitted from person-to-person.

b. Health care facilities are included in the types of buildings that have been associated with the transmission of *Legionella* to people. Cases of healthcare-associated LD (HCA LD) often arise from exposure to *Legionella* bacteria in hospital potable water distribution systems. The Centers for Disease Control and Prevention (CDC) guidance document on the prevention of healthcare-associated pneumonia defines laboratory-confirmed cases to be "definite" HCA LD if a patient has spent equal to or greater than 10 days continuously in a healthcare facility prior to the onset of LD, or "possible" HCA LD if a patient has spent 2 to 9 days in a healthcare facility prior to the onset of LD. ***NOTE:*** *In January 2014, the CDC National Center for Immunization and Respiratory Diseases released an updated Legionellosis Case Report form, including guidance on determining if the case was definitely or possibly associated with a healthcare exposure. VHA Directive 1061 focuses on primary prevention activities for buildings where patients, residents or visitors stay overnight; in the event of a LD case suspected to be associated with any VHA building, case investigation and consideration of secondary prevention activities would be appropriate.*

c. Persons at increased risk for LD include the immunocompromised (due to, for example, transplant, malignancy, renal disease, or diabetes), those over 50 years of age, those with chronic lung disease, and smokers. However, LD cases reported in the medical literature indicate that the disease can also occur in seemingly healthy individuals.

d. Given the various factors and complexities associated with LD (e.g., host susceptibility, pathogen virulence, water distribution system configurations and conditions), 100% prevention of LD is likely not possible. However, prevention and control practices can be implemented to reduce the risk of exposing people to *Legionella* in building water distribution systems. The *Legionella* prevention activities in this Directive involve assessing risks, monitoring water quality and implementation of commensurate engineering controls to limit the growth of *Legionella*. Use of engineering controls to limit *Legionella* growth includes ongoing monitoring of implemented controls, validating that the control measures are effective at inhibiting *Legionella* growth, and modifying implementation or type(s), as necessary. By focusing on engineering controls, this Directive can be viewed as a horizontal intervention that can improve the overall microbiological quality of facility water, not just the inhibition of *Legionella* growth.

e. *Legionella* growth in building potable water distribution systems is primarily suppressed by the implementation of engineering controls such as maintenance of appropriate water temperatures or biocide (e.g. residual oxidant) levels. Application of more than one control may be necessary for the successful inhibition of *Legionella* growth.

© 2018 Gamage SD et al. *JAMA Network Open.*

(1) **Maintenance of Appropriate Water Temperatures in Building Water Distribution Systems.**
(a) Water temperatures at 124 degrees Fahrenheit (°F) (51.1 degrees Celsius (°C)) or higher are necessary to inhibit *Legionella* growth in hot water systems.

1. For most adult individuals, 110°F at the water outlet (e.g., sink tap, showerhead) will minimize the risk of scalding and is consistent with the plumbing code adopted by the Department of Veterans Affairs (VA) for VHA buildings. At 117°F the risk of scalding increases significantly. At 140°F, second degree burns may occur after only 3 seconds of exposure. Some people, either due to illness, disabilities, extremes of age or side effects of medication, may be less sensitive to hot water temperatures or have impaired or reduced reactions and thus are at an increased risk for tissue damage caused by extended exposure to hot water.

2. It is not possible to maintain water temperatures at the outlet that kill *Legionella* bacteria and simultaneously eliminate the possibility of scald injury in persons partially or fully insensitive to hot water temperature or having delayed or impaired response capabilities. The water temperature and accompanying safety requirements in this Directive address the risk of inhalation or aspiration of live *Legionella* bacteria while minimizing the risk of scald injury from exposure to domestic hot water.

(b) Cold water systems (temperature at 67°F (19.4°C) or lower) tend to be too cold to foster growth of *Legionella*. Cold water in piping and fixtures can reach ambient environmental temperatures exceeding 68°F (20°C) during prolonged periods of low flow or non-use. Use of piping system insulation, automatic drain devices, and recirculation to limit the rate and duration of an increase in cold water temperature in combination with appropriate biocide levels can be effective at preventing *Legionella* growth.

(2) **Maintenance of Biocide at a Recommended Level for *Legionella* Control.**

(a) Minimum concentrations of various biocides (e.g. oxidizing agents such as chlorine) can inhibit the growth of *Legionella* in building potable water distribution systems. The use of one or more installed systemic water treatment system(s) may be necessary to supplement any residual disinfectant present in incoming water (from municipal or central plant sources). ***NOTE:*** *The United States (U.S.) Environmental Protection Agency (EPA) regulates contaminant levels and disinfectant treatment for use under the Safe Drinking Water Act (42 U.S.C. §§300f, et seq.). U.S. EPA delegates primacy to States for the regulation and enforcement of the Act within individual State boundaries if the standards set by the State are at least as stringent as EPA's.*

(b) The efficacy of biocides on suppressing or killing waterborne pathogens is dependent on multiple factors such as water quality, organic and inorganic contaminants, pH levels, water hardness, disinfectant concentrations, and contact time. Therefore, the minimum concentration of biocide necessary to suppress bacterial growth may vary from building to building and even within buildings.

© 2018 Gamage SD et al. *JAMA Network Open*.

f. Installation of non-systemic systems or processes has been used in some buildings as mechanisms to provide further control of *Legionella* growth or delivery.

g. This Directive was developed by the VA *Legionella* Expert Work Group, a multidisciplinary team consisting of subject matter experts from transplant centers, healthcare engineering, infectious diseases, pathology and laboratory medicine, infection prevention and control, construction and facilities management, public health, occupational safety and health, and healthcare operations.

3. POLICY: It is VHA policy that an ongoing program for HCA LD prevention, including provisions necessary for the prevention of scald injury, is implemented in all VHA buildings in which patients, residents or visitors stay overnight. This program must be established with written policy in accordance with, at a minimum, the requirements defined in this Directive.

4. RESPONSIBILITIES:

a. **Under Secretary for Health.** The Under Secretary for Health is responsible for:
(1) Establishing and providing resources for the national VHA Water Safety Program, with key subject matter expertise provided by the Office of Capital Asset Management, Engineering, and Support (OCAMES), and the National Infectious Diseases Service (NIDS).

(2) Authorizing the VHA Water Safety Program to conduct assessments and surveys related to implementation and ongoing monitoring of this Directive and prevention and control of waterborne pathogens, including *Legionella*, at VHA facilities.

(3) Authorizing the VHA Water Safety Program to develop and issue competency requirements, initial and continuing education, and additional requirements and guidelines for water safety.

b. **Deputy Under Secretary for Health for Operations and Management.** The Deputy Under Secretary for Health is responsible for:

(1) Appointing the VHA Water Safety Program Director; and

(2) Ensuring effective engagement of clinical and healthcare engineering resources.

c. **VHA Water Safety Program Director.** The VHA Water Safety Program Director is responsible for:

(1) Developing and issuing competency requirements, initial and continuing education, and any additional requirements and guidelines for the prevention of waterborne pathogens, including *Legionella*.

© 2018 Gamage SD et al. *JAMA Network Open*.

(2) Conducting assessments and surveys related to the implementation and ongoing monitoring of this Directive.

(3) Evaluating the reports submitted by each Veterans Integrated Service Network (VISN) regarding implementation of this Directive at VISN medical facilities.

(4) Providing consultative assistance to the VISNs and facilities, as needed.

(5) Defining VISN-level liaison responsibilities and competency requirements.

d. **VISN Director.** The VISN Director is responsible for:

(1) Ensuring that all facilities within the VISN comply with this Directive and any policies and guidance from the VHA Water Safety Program for prevention of HCA LD, prevention of scald injuries, and water safety.

(2) Prioritizing resources and support for implementation of this Directive for all facilities within the VISN.

(3) Ensuring completion of initial implementation and annual reporting requirements, completion of clinical and environmental testing, and subsequent submission of the reports to the VHA Water Safety Program.

(a) Providing the VHA Water Safety Program with progress reports on actions taken by facilities to meet the implementation requirements for every facility within the VISN.

(b) Providing the VHA Water Safety Program with progress reports on expected completion dates and supplemental actions taken to implement the engineering control strategies.

(c) Assigning a VISN-level staff member as the water safety liaison for communication between VHA Central Office and VISN or facility staff regarding water safety and *Legionella* prevention actions, policies, and guidance. The VISN-level liaison is expected to be knowledgeable in VHA policies and guidance for prevention of HCA LD, prevention of scald injuries, and water safety. **N*OTE:*** *Additional guidance regarding the VISN-level liaison responsibilities and competency requirements will be provided by the VHA Water Safety Program Director.*

e. **Medical Facility Director.** The medical facility Director is responsible for:
(1) Ensuring that the medical facility establishes a multi-disciplinary Facility Water Safety Committee no later than October 1, 2014. The Facility Water Safety Committee is required to be chaired by the medical facility Associate Director, or equivalent, and report to the medical facility Director. This committee must include, at a minimum, representation from the following areas: Engineering/Facilities Management, Infectious Diseases, Infection Prevention and Control, Pathology and Laboratory Medicine, Hemodialysis (if performed on site), Safety/Industrial Hygiene, and Occupational Health.

© 2018 Gamage SD et al. *JAMA Network Open*.

NOTE: *If the medical facility Director does not have a required member (e.g. Infectious Disease) then the medical facility Director should work with the VISN Director to identify a representative within the VISN to participate on the Facility Water Safety Committee. Other stakeholders in facility water use (e.g., labor partners, dental, sterile processing, and supply) may be included on the Facility Water Safety Committee, as appropriate.*

(2) Establishing a medical facility HCA LD prevention policy which specifies responsibilities and incorporates written HCA LD prevention plans no later than February 2, 2015.

(3) Ensuring that each building subject to this Directive has a written HCA LD prevention plan, including provisions necessary for the prevention of scald injury. The written plan(s) must be in compliance with the requirements of this Directive and any guidance issued by the VHA Water Safety Program Director.

(a) Each HCA LD prevention plan must be approved by the medical facility Director no later than December 31, 2014.

(b) The medical facility Director must certify that each building subject to this Directive has a written HCA LD prevention plan and approve the initial plan(s), and recertify annually thereafter.

(c) The medical facility Director must submit the initial and annual facility HCA LD prevention plan(s), certifications, and approvals to the VISN Director.

(4) Ensuring that the actions in the written HCA LD prevention plan(s) are implemented.

(5) Ensuring that the medical facility has decommissioned all indoor, open decorative water features from all of its buildings, and future design plans do not include the installation of such indoor, open water features. ***NOTE:*** *The use of indoor, open decorative water features has been epidemiologically-linked to LD in the healthcare setting.*

(6) Ensuring that each building subject to this Directive conducts at least quarterly environmental water testing for *Legionella* in accordance with this Directive, and submits the results to the VISN Director.

(7) Providing annually to the VISN Director a summary of the medical facility’s clinical *Legionella* testing results and number of cases of LD (definite HCA LD, possible HCA LD, and community-associated LD).

(8) Ensuring that all cases of LD are reported to the appropriate public health authority in accordance with applicable statutes, regulations, and with current VHA policy for infectious disease reporting, protecting health information, and release of information.

© 2018 Gamage SD et al. *JAMA Network Open*.

(9) Requesting consultative assistance from the VHA Water Safety Program, through the VISN-level water safety liaison, on issues related to *Legionella* implementation of prevention efforts, if needed.

f. **VHA Facility Chief of Staff and Associate Director of Patient Care Services.** The VHA facility Chief of Staff and Associate Director of Patient Care Services are responsible for:
(1) Ensuring that the medical facility has access to clinical care staff with expertise in infectious diseases to assist in diagnosis and treatment of LD. ***NOTE****: Diagnostic testing of pneumonia patients for LD, especially when healthcare-association is suspected, can provide important information for surveillance and remediation purposes. Full details on requirements and recommendations for clinical testing and diagnostic awareness can be found in Appendix C.*

(2) Ensuring that clinical staff involved in direct patient care are notified when cases of definite or possible HCA LD are identified to increase diagnostic awareness.

(3) Ensuring that clinical staff involved in direct patient care are notified when routine environmental water testing is positive for *Legionella* to increase diagnostic awareness (see Appendix C, paragraph 3 for more information).

g. **VHA Facility Chief Engineer or Facility Manager.** The VHA Facility Chief Engineer, Facility Manager, or equivalent is responsible for:
(1) Ensuring that the maintenance of appropriate water temperatures in the hot and cold potable water distribution system(s) is in accordance with Appendices A and B and the facility's approved HCA LD prevention plan(s).

(a) Documenting the facility's policy for the implementation and monitoring of temperature limits in the hot and cold potable water distribution systems (e.g., hot water tanks, if used, circulating water in the distribution systems, and at the outlets), including written explanation of any conditions or circumstances that may delay implementation of water temperatures, in accordance with Appendices A and B and the facility's approved HCA LD prevention plan(s).

(b) Preparing written documentation of engineering procedures according to the requirements in Appendix B for the prevention of scald injury.

(c) Ongoing monitoring of the temperature levels in the building's potable water distribution system(s) to ensure they are within the requirements defined in Appendices A and B and the facility's approved HCA LD prevention plan(s).

(d) Verifying that implementation and monitoring of water temperature levels is in accordance with the facility's written policy.

(2) Continuous monitoring of incoming water quality entering building(s) (from municipal or central plant sources) as required in Appendix A and the facility's approved HCA LD prevention plan(s).

(3) When a water treatment system(s) is present in a building to deliver a biocide (e.g. oxidant residual) into the system, ensuring the maintenance of appropriate biocide levels for *Legionella* control in the building's potable water distribution system(s) in accordance with Appendix A and in compliance with applicable regulatory requirements for safe drinking water and effluent concentrations.

(a) Biocide-based water treatment systems are subject to Federal and State statutes and regulations which typically identify acceptable biocide(s) and specify construction and operating requirements. Installed systems must be specifically approved or recognized for the intended use by the State regulatory water authority. Documentation of system(s) approval, design, installation, and operation shall be maintained current.

(b) Documentation of the facility's policy for biocide concentration levels in the hot and cold potable water distribution systems. This includes documentation of minimum and maximum biocide levels, allowable disinfection byproduct levels, biocide monitoring method and frequency, and any other requirements in accordance with Appendix A and in compliance with operating permits.

(c) Ongoing monitoring of biocide and disinfection byproduct levels in the building's potable water distribution systems to ensure they are within the guidelines defined in Appendix A and in compliance with operating permit requirements.

(4) Ensuring that any treatment measures implemented in building water distribution systems are functioning according to the manufacturer's specifications for the particular system that is being used and at recommended capacity for *Legionella* inhibition.

(5) Conducting Infection Control Risk Assessments in cooperation with other facility stakeholders to address the potential impact of construction and maintenance of water systems on growth or transmission of waterborne pathogens and to determine the extent of precautions, disinfection, and system or component commissioning requirements.

(6) Ensuring that newly installed piping and distribution system components are flushed of debris and disinfected prior to being placed into service. Piping and components must be cleaned and protected from accumulation of debris and contamination prior to and during installation. Documentation of flushing and disinfection must be maintained.
(7) Ensuring unused water branch lines and dead-legs are removed and capped at the main supply/recirculation supply lines to limit stagnation and reservoirs for *Legionella* growth in accordance with the approved HCA LD plan(s).

(8) Ensuring that only steam is used for building humidification purposes. Comply with prohibition of ultrasonic humidifiers, foggers, misters, spray humidifiers, and tank type humidifiers.

© 2018 Gamage SD et al. *JAMA Network Open*.

(9) Assessing and documenting competency of contractors and the contractor's personnel as part of the acquisition process prior to the start of any work on facility water systems, including water treatment. Competencies must be re-assessed on an on-going basis, or whenever there is a change in contractors or the contractor's personnel performing the work. At a minimum, the contractor's competency should be assessed and documented on an annual basis. A copy of any assessment or documentation must be submitted to the supporting VISN Contract Manager.

(10) Ensuring competent personnel are available at all times to address water system operations.

(11) Forwarding notification to all medical facility employees when:

(a) Maintenance and repair procedures will be taking place that could affect the water system;

(b) Maintenance and repair procedures have been completed; and

(c) Affected systems have been tested and are returned to normal operation.

(12) Providing the Facility Water Safety Committee, Safety Committee, and Infection Control Committee with an annual report of the water system maintenance and monitoring and any *Legionella* mitigation actions taken.

h. **VHA Facility Chief of Pathology and Laboratory Medicine.** The VHA Facility Chief of Pathology and Laboratory Medicine Service is responsible for:
(1) Ensuring that the laboratory has access to *L. pneumophila* urinary antigen testing. VHA-designated Transplant Centers need to consider on-site availability of *L. pneumophila* urinary antigen testing.

(2) Ensuring access to a clinical laboratory that can perform cultures on respiratory secretions for *Legionella*, with identification at the species level, and can determine the serogroup of *L. pneumophila.*

(3) Ensuring that clinical cultures for *Legionella* and urinary antigen tests are performed in accordance with current VHA policy on laboratory testing.

(4) Ensuring that results from laboratory tests and clinical cultures for *Legionella* are entered into the Computerized Patient Record System (CPRS) as soon as testing is completed.

(5) Providing annually to the Infection Control Committee and the Facility Water Safety Committee the:

(a) Total number of urinary antigen tests and clinical cultures for *Legionella* ordered; and

© 2018 Gamage SD et al. *JAMA Network Open*.

(b) Total number of persons with positive results for *Legionella.*

(6) Ensuring that the Laboratory Service is involved in the process of selecting a laboratory that can perform environmental water and swab cultures for *Legionella* (see Appendix C, paragraph 2 for requirements and guidance on selecting an environmental testing laboratory).

i. **Facility Water Safety Committee Chair.** The Facility Water Safety Committee Chair is responsible for:
(1) Conducting an annual assessment to determine which buildings fall subject to this Directive.

(2) Developing written HCA LD prevention plan(s) for each building subject to this Directive in accordance with this Directive and requirements promulgated by the VHA Water Safety Program. The written HCA LD prevention plan(s) must contain, at minimum, all of the components delineated in Appendix A, paragraph 1. These components address building associated risk assessments, implementation and monitoring of engineering controls (see Appendix A and B for details), validation that the engineering controls are effectively preventing *Legionella* growth (see Appendix C for details), and scald prevention.

(3) Reviewing the written HCA LD prevention plan(s) at least annually and updating as necessary.

(4) For each building subject to this Directive, establishing the policy for conducting environmental water testing for *Legionella*, to include:

(a) Determining the number and location of outlets tested and the frequency of such testing, which must be in accordance with at least the requirements in Appendix C, paragraph 2.a.

(b) Determining who at the building level is responsible for: collecting environmental water samples, ensuring that the water samples are transferred to the environmental testing laboratory, receiving the results, and reporting the results to the Facility Water Safety Committee, Safety Committee and the Infection Control Committee.

(c) Determining the laboratory that will conduct the environmental water testing using the requirements and guidance in Appendix C, paragraph 2.b.

(5) Conducting routine meetings to review the records from implementation of the HCA LD prevention plan(s).

(a) Routine committee meetings must be held at least quarterly. The reviews must assess, at a minimum, building associated risk(s), documented verification of policy implementation (e.g., implementation of engineering controls, water quality testing, water

© 2018 Gamage SD et al. *JAMA Network Open.*

pressure, scald control), any results from water testing for *Legionella*, whether any engineering controls were not within specified limits and why that may have occurred, whether any corrective actions were taken on engineering controls, whether the HCA LD prevention plan(s) needs to be updated, and whether there have been any cases of LD diagnosed at or potentially associated with each building.
NOTE: *If the Facility Water Safety Committee discusses any case of LD, patient information must be de-identified prior to sharing the information with the committee.*

(b) The Facility Water Safety Committee is to meet as necessary to address any non-routine *Legionella* control issues and HCA LD.

(c) Documenting in the minutes any corrective actions that were initiated for maintaining water temperature and oxidant residual at appropriate levels to inhibit *Legionella* growth, and documenting the effectiveness of the corrective actions taken.

(d) Ensuring the results of the reviews are communicated to the medical facility Leadership Team, Safety Committee, Infection Control Committee, and any other local committees as appropriate for the medical facility.

5. REFERENCES:

a. American Society for Heating, Refrigerating and Air-conditioning Engineers (ASHRAE). Guideline 12-2000. Minimizing the Risk of Legionellosis Associated with Building Water Systems; 2000.

b. American Thoracic Society and Infectious Diseases Society of America. Guidelines for the Management of Adults with Hospital-acquired, Ventilator-associated, and Healthcare-associated Pneumonia. American Journal of Respiratory and Critical Care Medicine. 171(4):388-416; 2005.

c. Association of Water Technologies. *Legionella* 2003: An Update and Statement by the Association of Water Technologies (AWT); June, 2003. http://awt.org/IndustryResources/*Legionella*03.pdf.

d. Centers for Disease Control and Prevention. Guidelines for Environmental Infection Control in Health-care Facilities. Recommendations of CDC and the Healthcare Infection Control Practices Advisory Committee (HICPAC). Morbidity and Mortality Weekly Reports. 52 (RR10):1-42; 2003. www.cdc.gov/mmwr/preview/mmwrhtml/rr5210a1.htm.

e. Centers for Disease Control and Prevention. Guidelines for Preventing Healthcare-associated Pneumonia. Recommendations of CDC and the Healthcare Infection Control Practices Advisory Committee. Morbidity and Mortality Weekly Reports. 53(RR03):1-36; 2003. www.cdc.gov/mmwr/preview/mmwrhtml/rr5303a1.htm.

f. Department of Veterans Affairs Office of Inspector General. Assessment of Legionnaire's Disease Risk in Veterans Health Administration Inpatient Facilities. Report

© 2018 Gamage SD et al. *JAMA Network Open*.

No. 07-00029-151; June 20, 2007. http://www.va.gov/oig/54/reports/VAOIG-07-00029-151.pdf.

g. Department of Veterans Affairs Office of Inspector General. Healthcare Inspection: Prevention of Legionnaires' Disease in VHA Facilities. Report No. 13-01189-267; August 1, 2013. http://www.va.gov/oig/pubs/VAOIG-13-01189-267.pdf.

h. Department of Veterans Affairs, Veterans Health Administration, Executive Decision Memorandum. Indoor Decorative Water Features in VHA Healthcare Facilities. Approved November 26, 2012. Located at http://vaww.nlc.portal.va.gov/EDMs/EDMDocuments/2012%20Approved%20Executive%20Decision%20Memos/EDM%20Indoor%20Decorative%20Water%20Features.pdf. ***NOTE:*** *This is an internal VA Web site and is not available to the public.*

i. Facilities Guidelines Institute. Guidelines for Design and Construction of Health Care Facilities, 2010 edition. Chicago: American Society of Healthcare Engineering of the American Hospital Association; 2010.
j. International Code Council. International Plumbing Code (IPC), Chapters 1 through 6 2009 IPC. International Code Council, Inc.; 2009.

k. Mandell LA, Wunderink RG, Anzueto A, Bartlett JG, Campbell GD, et al. Infectious Diseases Society of America/American Thoracic Society Consensus Guidelines on the Management of Community-Acquired Pneumonia in Adults. *Clinical Infectious Diseases* 44:S27-72; 2007. l. Occupational Safety & Health Administration (OSHA). OSHA Technical Manual, Section III: Chapter 7. Legionnaire's Disease. Effective date: January 20, 1999.

l. World Health Organization (WHO). *Legionella* and the Prevention of Legionellosis. WHO Press; 2007. http://www.who.int/water_sanitation_health/emerging/*Legionella*.pdf.

m. 40 CFR part141. National Primary Drinking Water Regulations.

n. 40 CFR part142. National Primary Drinking Water Regulations Implementation.

6. DEFINITIONS:
a. **Anti-scald device.** Anti-scald device is a temperature actuated appurtenance used in plumbing systems to reduce/stop water flow exceeding a defined temperature. Individual anti-scald devices must meet American Society of Sanitary Engineers (ASSE) 1062 Standard. Anti-scald devices may be add-on or integrated into plumbing fixtures or integrated into water tempering valves.
b. **Biocide.** For the purpose of this Directive, a biocide is a chemical agent or substance which can deter, inactivate, or kill microorganisms.
c. **Chlorine.** Chlorine is an EPA listed biocide chemical (oxidant) approved for use in the treatment of potable water to control/inactivate waterborne bacteria, viruses, and

protozoa. In high concentrations, it can be used for the disinfection of water systems and their components.

d. **Chlorine dioxide.** Chlorine dioxide is an EPA listed biocide chemical (oxidant) approved for use in the treatment of potable water to control/inactivate waterborne bacteria, viruses, and protozoa.

e. **Clinical testing.** Clinical testing encompasses the spectrum of diagnostic modalities that are used to elucidate the cause of a disease process. In particular, for this Directive, they are

specific for the diagnostic modalities used to determine the presence of LD. These clinical testing modalities include culture for *Legionella* species, *Legionella pneumophila* urinary antigen testing, serological testing using IgG and IgM antibodies with acute and convalescent serology (convalescent serology is obtained "after the fact" and can help confirm a diagnosis in retrospect), and nucleic acid antibody/molecular diagnostic testing. There is an expectation that appropriate clinical testing is done as standard practice by providers when caring for patients with a certain disease process.

f. **Clinical Validation.** Clinical validation is the process of determining if the primary engineering controls and any supplemental water treatment systems are successfully inhibiting *Legionella* growth in the potable water distribution system(s) by monitoring the occurrence of HCA LD. For the purpose of this Directive, clinical validation encompasses diagnostic testing of HCA pneumonia cases for LD when indicated and heightened awareness for diagnostic testing when *Legionella* is detected in environmental samples.

g. **Community-associated *Legionella* disease (CA-LD).** In general, CA-LD is a laboratory-confirmed case of *Legionella* disease in which the patient has not had contact with the healthcare setting in the 10 days prior to onset of illness.

h. **Control measure.** A control measure is any action or activity that can be used to prevent or eliminate a hazard or reduce the hazard to an acceptable level. [WHO, 2007].

i. **Continual or Continuous.** For the purposes of this Directive, continual or continuous refers to the almost uninterrupted monitoring and control of: water quality, incoming water pressure, biocide levels, and water temperatures. This can be achieved through the use of automated measurement/control devices, typically connected to the Building Automation System (BAS), at various locations such as water source equipment and throughout the water distribution system. Central recording of measurements is needed at least every 30 minutes and instantaneously during out-of-control limit (alarm) conditions. More frequent measurement recording for some or all parameters/locations may be required by the Facility Water Safety Committee or the State (or its delegated local water authority).

j. **Corrective action.** Corrective action is any action to be taken when the results of monitoring indicate that a primary or supplemental control is not within the established control limits.

k. **Disinfection.** Disinfection is an irreversible inactivation of microorganisms on a surface or in a system and the reduction of those microorganisms to non-hazardous levels. Low level chemical disinfection of potable water systems can be accomplished through the use of EPA listed biocides. High level disinfection for new installations or maintenance of piping, equipment, and components is conducted in accordance with the

© 2018 Gamage SD et al. *JAMA Network Open*.

requirements of the International Plumbing Code (IPC 2009), American Water Works Association (AWWA C651-05), and VA Master Construction Specifications.

l. **Distal.** Distal means remote. In plumbing systems "distal" piping, equipment, or water outlets can be either physically or hydraulically remote, or in instances both, from a source point. ***NOTE:*** *The term "distal water site" was used in previous VHA Legionella prevention policy to*

refer to points where the end user accesses the water; this term has been replaced by "outlet" in this Directive.

m. **Emergency Remediation.** Emergency Remediation is the process of implementing immediate, temporary actions to reduce the amount of *Legionella* in a water distribution system.

n. **Engineering Control limit.** An engineering control limit is a minimum and or maximum value at which a parameter must be maintained in order to prevent or eliminate a hazard or reduce the hazard to an acceptable level. For example, a minimum hot water temperature of 124 degrees Fahrenheit (°F) inhibits *Legionella* growth in building hot water systems.

o. **Environmental Validation.** For the purpose of this Directive, environmental validation is the process of testing the building's potable water distribution system(s) to determine if engineering controls are successfully inhibiting growth of *Legionella*.

p. **Flushing of Outlets.** Flushing of outlets is the process of opening outlets such that hot and cold water flows out of the outlet for a specified period of time (specific duration varies with purpose and process). The purpose of routine flushing is to prevent stagnating conditions in pipes which could result in tempering of water temperature, dissipation of biocide, and establishment of favorable conditions for *Legionella* growth.

q. **Healthcare-associated *Legionella* disease (HCA LD).** HCA LD is a laboratory-confirmed case of *Legionella* disease that is epidemiologically-linked to the healthcare facility. HCA LD cases may be "definite" or "possible". The definitions below for these classifications are based on information in the CDC 2003 guidelines for prevention of healthcare-associated pneumonia and the updated CDC Legionellosis Case Report form released in January, 2014. ***NOTE:*** *Each case of LD should be assessed for linkage to the facility on a case-by-case basis taking into account any related factors (e.g., a change in definition during a LD outbreak, molecular matching of patient and environmental isolates).*

(1) **Definite HCA LD.** "Definite HCA LD" is a laboratory-confirmed case of *Legionella* disease with an inpatient stay that is equal to or greater than 10 days of continuous inpatient stay prior to onset of illness. [CDC Guidelines, 2003; CDC Legionellosis Case Report form, 2014]

(2) **Possible HCA LD.**

(a) "Possible HCA LD (inpatient)" is a laboratory-confirmed case of *Legionella* disease in which a patient has spent 2 to 9 days in a healthcare facility prior to onset of illness. [CDC Guidelines, 2003]

(b) "Possible HCA LD (inpatient and outpatient)" is a laboratory-confirmed case of *Legionella* disease in which the patient had exposure to a healthcare facility for a portion of the 10 days prior to onset of illness. [CDC Legionellosis Case Report form, 2014]

r. **Healthcare-associated *Legionella* Disease (HCA LD) Prevention Plan.** The HCA LD Prevention Plan is the written plan required for every VHA building where patients,

© 2018 Gamage SD et al. *JAMA Network Open*.

residents, or visitors stay overnight. The HCA LD Prevention Plan focuses on identification of risks and
implementation of engineering measures for control of *Legionella* growth, monitoring of the control measures, validation that the measures are effective at suppressing *Legionella* growth, and implementation of corrective actions when indicated. The HCA LD Prevention Plan is to be reviewed at least annually for any updating.

s. **Hyperchlorination.** See "Shock chlorination".

t. **Immersion Bath.** Immersion bath is a bath in which an individual's entire body or a body part is submerged in water.

u. ***Legionella*.** *Legionella* is a Gram-negative bacterium that is naturally found in water and has been associated with building water distribution systems and cooling towers. Over 50 species and 70 serogroups have been identified.

v. ***Legionella* Disease (LD).** LD is the term used in this Directive for the disease traditionally known as "Legionnaires' disease;" a type of pneumonia caused by pathogenic species of the bacterium, *Legionella*. Most, but not all, cases of disease are caused by the species *Legionella pneumophila* serogroup 1.

w. **Legionellosis.** Legionellosis refers to diseases (Legionnaires' disease, Pontiac Fever) caused by pathogenic species of *Legionella* bacteria. Legionnaires' disease is defined above (see "*Legionella* disease"). Pontiac fever is a milder respiratory infection and symptoms resolve without treatment.

x. **Legionnaires' Disease.** See "*Legionella* disease."

y. **Mitigation.** Mitigation is a process of implementing actions to reduce the amount of *Legionella* in a water distribution system (also called "remediation").

z. **Mixing Valve.** Mixing valve is a generic reference to a class of water tempering devices. Mixing valves used for tempering hot water in potable water systems must meet the requirements of the International Plumbing Code (IPC 2009), American Society of Sanitary Engineers (ASSE 1016/1069/1070), and VA Master Construction Specifications.

aa. **Monitoring.** Monitoring, for the purpose of this Directive, refers to the process of routinely checking water quality of incoming water (from municipal or central plant) and implementation of primary and any supplemental engineering controls to determine if the controls are within established minimum and maximum limits.

bb. **Monochloramine.** Monochloramine is a type of chloramine. Chloramines are most commonly formed when ammonia is added to chlorine. An EPA listed biocide chemical (oxidant) approved for use in treatment of potable water to control/inactivate waterborne bacteria, viruses, and protozoa.

cc. **Outlet.** Outlet is a point in the potable water distribution system where the individual (also known as the "end user") accesses the water. Examples include faucets, showers, ice machines, and drinking fountains. ***NOTE:*** *In previous VHA Legionella prevention Directives, the term "distal site" was used instead of "outlet."*

dd. **Oxidant residual.** Oxidant residual is the amount of available oxidant present in the water system and at the outlet after demand has been satisfied. Required oxidant residuals in a water system are, in-part, determined by: water quality, water system conditions (piping system corrosion, water flow/turnover, etc.), presence of disinfection by-products, and type and quantity of microorganisms requiring inactivation.

ee. **Point-of-use filter.** Point-of-use filter is a micropore filter specifically designed for use in preventing the passage of *Legionella* bacteria, other specific microorganisms, and

© 2018 Gamage SD et al. *JAMA Network Open.*

particle contaminants present in water. Typically, these filters are fitted to water outlets or installed in water supply lines proximal to equipment (e.g., ice machines, drinking fountains).

ff. **Potable Water Distribution System.** Potable water distribution system is a water distribution system (hot water and cold [unheated] water) within a building or structure primarily used for drinking, sanitation, food service, and personal hygiene meeting EPA and state drinking water standards.

gg. **Primary control measures.** Primary control measure refers to the main or routine methods used to suppress *Legionella* growth in building potable water distribution systems. Primary control measures often used in building potable water distribution systems include at least one of the following: appropriate water temperature(s) and treatment with biocide(s) (e.g. oxidizing agent).

hh. **Process flow diagram.** Process flow diagram is a systematic representation of the sequence of steps or operations used in the production or manufacture of particular item [WHO, 2007].

ii. **Remediation.** Remediation is the process of implementing actions to reduce the amount of *Legionella* in a water distribution system (also called "mitigation").

jj. **Resident.** Resident is defined here for the purpose of clarifying which buildings fall subject to this Directive. In the phrase "VHA buildings in which patients, residents or visitors stay overnight", the term "resident" refers to Veterans who are under residential-type care such as provided at a Community Living Center or domiciliary.

kk. **Shock chlorination.** Shock chlorination is the application of hypochlorite, usually in the form of a solution, to the water distribution system at higher than normal levels for remediation purposes.

ll. **Supplemental or Supplementary Water Treatment.** A measure used in addition to routine (primary) control measures to inhibit the growth of *Legionella* in building water distribution systems.

mm. **Thermal eradication.** Thermal eradication is the temporary resetting of the temperature in the hot water distribution system to 160°F - 170°F (71°C - 77°C) while continuously flushing each outlet in the system for at least 30 minutes (also known as "super heat and flush").

nn. **Validation.** Validation is the process of obtaining evidence that a plan is effective. For purposes of this Directive, validation specifically refers to verifying that the primary and any supplemental engineering controls are effective at inhibiting the growth of *Legionella* in building potable water distribution systems. The two validation methods used are environmental water testing for *Legionella* and clinical testing of pneumonia patients for *Legionella*.

oo. **Water distribution system.** Water distribution system is a system used for the distribution of potable water (site and building) which includes all piping, water treatment, equipment, controls, fixtures, and components.

pp. **Water System Management Point.** A specific position, device, fixture, or water distribution system component used for the monitoring of conditions or performance or the control of the system or its individual components.

© 2018 Gamage SD et al. *JAMA Network Open*.

APPENDIX A
PREVENTION PLANS FOR HEALTHCARE-ASSOCIATED (HCA)
***LEGIONELLA* DISEASE (LD)**

1. COMPONENTS OF A HCA LD PREVENTION PLAN: A HCA LD prevention plan must include:

a. Schematic (single line) diagrams of the site distribution and domestic water systems (hot and cold). Each diagram must be kept current and include diagrams of how water is distributed, circulated, stored, heated and cooled, treated, and monitored. The diagrams must be accurate representations of existing conditions and focus on main areas of water distribution and processing system(s) and identify any areas in which water is processed differently (e.g., hemodialysis and sterile processing). Further information on what comprises a schematic diagram will be provided by the VHA Water Safety Program.

b. A risk assessment of the building for HCA LD. At least annually, assess the building for factors that may indicate increased risk for HCA LD. Factors can include, but are not limited to: patient population risk factors, presence of building units associated with increased risk (e.g. transplant units), past cases of HCA LD, ability to implement engineering controls to prevent *Legionella* growth, past positive environmental testing results, and location of the building in an area of the country with recognized higher incidence of LD. Implementation of previous years' HCA LD plans and their findings should also be included in the risk assessment. ***NOTE:*** *This risk assessment can be useful when implementing the validation activities in Appendix C and determining follow-up actions.*

c. Identification of water system management points for the building's potable water distribution system(s), and, based on the schematic diagrams, where monitoring and controls can be implemented to prevent the growth of *Legionella* and prevent scald injury.

d. Establishment of engineering control strategies. Specifically, the HCA LD Prevention Plan needs to:

(1) Establish the engineering control limits for each strategy to inhibit *Legionella* in the environment (see paragraph 2 below).

(2) Identify control mechanisms for preventing scald injury from water that is too hot (see Appendix B).

(3) Establish a schedule to routinely monitor implementation of the control strategies. ***NOTE:*** *Since this Directive focuses on the implementation of engineering controls to prevent Legionella growth, "monitoring" refers to assessment of the levels of the control measures (e.g., water temperature, biocide level) in the water distribution system and water quality, not the amount of Legionella. Rather, assessment of Legionella in the*

© 2018 Gamage SD et al. *JAMA Network Open*.

water distribution must be included as a mechanism to validate that the engineering controls are effective.

(4) Establish a dead-leg elimination and prevention plan. Plan components include: identification of existing dead-legs, dead-leg risk assessment, removal prioritization, removal schedule, and prevention. e. Documenting when each water quality and control measure was monitored for condition compliance and corrective action taken (what and when).

f. Validation that the control measures are effectively inhibiting *Legionella* growth (see Appendix C).

g. Process flow diagrams of the different control strategies and monitoring for each building's hot and cold water distribution systems. ***NOTE:*** *Each diagram is to focus on main areas of water distribution and processing and identify any areas in which water is processed differently (e.g., hemodialysis and sterile processing).*

2. ENGINEERING CONTROL STRATEGIES AND LIMITS FOR ONGOING PREVENTION OF *LEGIONELLA* GROWTH: Maintenance of appropriate water temperatures and implementation of biocide, if indicated, comprise the primary control measures used to inhibit *Legionella* growth in the potable water distribution systems of buildings where patients, residents or visitors experience an overnight stay. The building's potable water distribution system(s) is to be maintained and monitored in accordance with the following requirements:

a. **Water Quality and Pressure Monitoring.** Potable water entering each building subject to this Directive shall be continuously monitored for incoming water pressure and the following characteristics: temperature, pH, dissolved solids, and oxidant residual. ***NOTE:*** *Dependent upon local conditions and whether water treatment systems are installed and operated, additional monitoring of water characteristics and contaminants may be required.*

b. **Water Temperature.** VHA requirements for water temperature limits for *Legionella* control in the building's potable hot and cold water distribution systems are as follows:

(1) **Hot Water Distribution Systems.** If a building uses domestic hot water storage tanks, water temperature of all such storage tanks must be maintained at a minimum of 140 degrees Fahrenheit (°F) (60 degrees Celsius (°C)) to prevent *Legionella* growth. The minimum discharge temperature for instantaneous and semi-instantaneous heat exchangers must be 130°F (54.4°C). Water in the potable hot water distribution system piping must be no lower than 124°F (51.1°C) (prior to any temperature-reducing mixing valve or anti-scald device at the water outlet). ***NOTE:*** *To limit the risk of scald injury, hot water in the distribution system piping should be maintained at the lowest temperature that will ensure the minimum of 124°F (51.1°C) throughout.*

© 2018 Gamage SD et al. *JAMA Network Open*.

(2) **Cold Water Distribution Systems.** *Legionella* can grow in the building's cold water distribution system as water temperatures increase above 67°F (19.4°C). Cold water temperature throughout the system should be maintained at or below 67°F (19.4°C) to the greatest extent practicable to inhibit growth. ***NOTE:*** *Use of piping system insulation, automatic drain devices and recirculation can limit the rate and duration of increased temperatures within the cold water distribution system. Based on local conditions and validation testing, modifications, upgrades and supplemental cooling of the cold water distribution system may be required.*

(3) **Water Temperature Monitoring.** The water temperature in the hot and cold potable water distribution systems needs to be monitored continuously to determine if temperatures are within the established control limits. Temperature monitoring must be conducted, at a minimum, in the following types of areas: incoming water supply to the building, water storage tanks, hot water discharge at the hot water source equipment, hot water return proximal to the hot water source equipment, water at the return of circulation loops, and water supplied to representative outlets (e.g., loop or branch, hydraulic remoteness, flow).

(4) **Water Temperature Control at the Outlet.** Buildings subject to this Directive must minimize the risk of scald injury to patients, residents, staff and visitors. The use of mixing valves and anti-scald devices on all outlets where people access water from the potable hot water distribution system is required in order to prevent scald injury. The water temperature delivered from the outlet must not exceed 110°F (43.3°C). See Appendix B for specific requirements and guidelines for the prevention of scald injury.

c. **Biocide.** Oxidizing agents have long been utilized by municipal water treatment facilities for inhibiting bacterial growth in public water supplies. Implementing systems to deliver oxidizing agents and other biocides in building water distribution systems can be effective in inhibiting bacterial growth; however, their operation requires careful oversight for effective and safe use. For all buildings subject to this Directive, the following section includes the requirement to assess the quality of incoming water, and guidance and recommendations in the event that a VHA medical facility decides to implement biocide-based water treatment system(s) in such buildings.

(1) Oxidant residual levels in the building incoming water supply and at representative outlets must be assessed. This assessment will determine if any disinfectant water treatment from the municipality or other potable water source is present when the water reaches the building and after distribution in the building. Knowing these values will aid in determining if oxidant residual levels are at a sufficient level to suppress *Legionella* growth (if present) and will contribute to the information available if deciding whether or not to install a treatment system(s).

(a) Monitoring the oxidant residual level in the incoming water supply is to be continual. Monitoring the oxidant residual in water supplied to representative outlets (e.g., loop or branch, hydraulic remoteness, flow) is to be continual.

© 2018 Gamage SD et al. *JAMA Network Open.*

(b) Minimum concentrations of oxidant residual necessary for inhibition of *Legionella* growth may vary from building to building. In general, the following minimum detected oxidant residual levels at hot and cold water outlets are suggested as guidance: 0.5 milligrams (mg) per liter (L) for chlorine (as free chlorine), 0.5 mg/L for monochloramine, and 0.3 mg/L for chlorine dioxide. ***NOTE:*** *These concentrations are considered guidance. Facilities may find that higher or lower levels are needed for Legionella growth inhibition in their building(s) based on local conditions and environmental testing for Legionella.*

(2) Facilities may choose to implement a systemic supplementary water treatment system(s) in buildings to supplement municipal or source treatment of water. Factors to consider for this decision include, but are not limited to: the levels of oxidant residual in the incoming water supply and/or at outlets, past history of HCA LD, and results from environmental and clinical validation testing (see Appendix C). If the facility decides to install a supplemental water treatment system in a building, then the following actions are required:

(a) Any biocides for use in systemic water treatment systems must be specifically approved or recognized for the intended use by the State regulatory water authority. VHA recognizes U.S. Environmental Protection Agency approved oxidants (chlorine, monochloramine, and chlorine dioxide) as acceptable disinfectants for use in potable water distribution systems. Use of an alternative biocide is permitted if the medical facility obtains a waiver (subject to its conditions and duration). ***NOTE:*** *Waiver process information and requirements will be provided by the VHA Water Safety Program.*

(b) The Facility Water Safety Committee must determine the appropriate type of supplemental water treatment system for the building. The facility must consult with the State (or its delegated local water authority) for regulating drinking water for guidance on system selection, achieving an appropriate biocide residual level at building outlets for *Legionella* growth suppression, system design, system operation, and ensuring compliance with regulations regarding water treatment system(s) and safety. Once a type of system is selected, either the State (or its delegated local water authority) or the manufacturer of the system must provide the minimum and maximum outlet biocide levels in writing for both hot and cold water. ***NOTE:*** *See paragraph 5 below regarding special-use water systems.*

(c) Biocide Residual Monitoring. The biocide residual levels of the water at distal water outlets in the hot and cold potable water distribution systems needs to be monitored to determine if levels are within the established control limits and in compliance with regulatory requirements. In addition, comply with regulatory requirements for contaminant monitoring frequency and locations.

d. **Flushing.** Regular flushing of hot and cold water at outlets (e.g., sink taps, showers), particularly those not in routine use or which experience low water flow, is necessary to ensure that engineering controls are maintained at sufficient levels for *Legionella* growth inhibition throughout the water distribution systems and at fixtures. Irregular use or low

© 2018 Gamage SD et al. *JAMA Network Open*.

flow fixtures must be flushed at least twice per week to prevent water stagnation for extended periods of time.

e. **Corrective Actions.** If routine monitoring determines that the water temperatures or biocide residual levels from an installed system are not within the established limits, then the following actions, at a minimum, must occur:

(1) Assess the reason(s) why the control(s) were not within the established limit.

(2) Corrective actions must be undertaken promptly, based on the assessment, to satisfy implementation of the control measures within established limits.

(3) Re-assess the controls measures after corrective actions are implemented to determine if the water system management point is within the established parameters. If not within the established parameters, reassess the corrective actions, and implement revised corrective actions.

f. **Documentation.** Water temperature and biocide residual testing, as well as corrective actions, must be documented to provide verification of implementation and monitoring.

g. **Progress Reporting.** After March 2, 2015 and then annually, the VISN Director must provide the VHA Water Safety Program with a progress report on actions taken to meet the implementation requirements for every medical facility within the VISN. If the engineering controls (i.e., water temperature and biocide treatment system, if chosen) are not implemented fully, the VISN Director's progress report(s) are to be submitted semi-annually and must include any expected completion dates and supplemental actions taken (See paragraph 4 below).

h. **Validation of *Legionella* Prevention.** Validation focuses on collecting and evaluating information to determine if the engineering controls are effectively controlling *Legionella* growth in the building's potable water distribution systems. See Appendix C for the validation requirements, which include both a clinical component to assess incidence of HCA LD and an environmental component to assess the presence of *Legionella* in the water distribution system.

3. SUPPLEMENTAL ACTIONS: Until the primary prevention strategy (i.e., water temperature and biocide treatment system, if chosen) is implemented fully, supplemental actions may be necessary to prevent and/or assess *Legionella* growth in building water distribution systems based on local conditions and validation results. ***NOTE:*** *If a building meets the primary prevention requirements, the medical facility may also choose to implement these measures based on local considerations.*

a. **Environmental Water Testing for *Legionella*.** Facilities may consider increasing the frequency of environmental water testing for *Legionella* during the year beyond the testing frequency required in this Directive (see Appendix C) based on local risk assessment (e.g., history of HCA LD, patient population, ability to implement

© 2018 Gamage SD et al. *JAMA Network Open.*

engineering controls) to determine if additional control procedures need to be implemented.

b. **Supplementary Water Treatment Measures.** Supplementary treatment measures that suppress *Legionella* growth and minimize the risk of exposure may be ongoing and systemic, immediate and systemic, or directed at a certain portion of the water distribution system. Supplementary water treatment measures must be maintained according to manufacturer's specifications and in strict compliance with State regulations and operating permits. Supplementary water treatment measures need to be identified in the process flow diagrams and control limits identified. The systems must be monitored and adjusted in a timely manner, if indicated, to ensure operation at a capacity to inhibit the growth of *Legionella*. Documentation of system verification and maintenance activities is required.

c. **Point-of-use Filters.** Point-of-use filters may be installed at specific outlets to prevent *Legionella* exposure to patients. This method may be of particular use in areas that treat high-risk patients.

4. EMERGENCY REMEDIATION OF THE POTABLE WATER DISTRIBUTION SYSTEMS: Emergency remediation of a building's potable water distribution system(s) is triggered, at a minimum, by certain occurrences: identification of a definite HCA LD case, identification of a possible HCA LD case and *Legionella*-positive water results, or identification of *Legionella*-positive water results during routine environmental testing. See Appendix C (Clinical and Environmental Validation of Primary Engineering Controls for Prevention of *Legionella* Growth) for specific requirements and detailed information for assessing when emergency remediation is to be conducted.

a. Emergency remediation is to include any or all of the following immediate procedures:

(1) **Thermal Eradication.** This procedure involves the temporary resetting of the temperature in the hot water distribution system(s) to 160 °F - 170°F (71°C - 77°C) while continuously flushing each outlet in the system for at least 30 minutes. Consideration needs to be given as to the feasibility of implementing thermal eradication depending on the design of the mixing valves in place. ***NOTE:*** *Since there is significant risk for scalding at the water temperatures used for thermal eradication, extreme care must be taken to protect end users of the water distribution system(s), as well as employees who are administering the measure.*

(2) **Shock chlorination.** This method involves increasing the chlorine level of the hot and cold water distribution systems to at least 2 mg/L and maintaining that level throughout the systems for at least 2 hours (but not exceeding 24 hours) and flushing all outlets. Chlorination of the hot water tank(s) or the water heater(s) to a concentration of 20 to 50 mg/L may be required to achieve this level of free chlorine residual. After the shock chlorination procedure is complete, the system must be thoroughly flushed before reuse. If post-shock chlorination water testing indicates that *Legionella* bacteria are still present in the water distribution system(s), it may be necessary to repeat shock chlorination with

© 2018 Gamage SD et al. *JAMA Network Open*.

consideration for use of a higher concentration of chlorine (e.g., at least 10 mg/ml free chlorine residual throughout the system and at outlets for 24 hours or 200 mg/L for three hours; refer to **Disinfection** definition). ***NOTE:*** *Consultation with the VHA Water Safety Program is strongly recommended prior to implementing shock chlorination with higher concentrations of chlorine.*

b. Thermal eradication and shock chlorination are temporary measures. After emergency mitigation, perform environmental testing to determine the effectiveness of the mitigation action. *Legionella* will likely reappear if proper routine water temperatures or residual biocide levels (or other supplementary systems or processes) are not maintained.

c. Prior to the implementation of emergency mitigation, stakeholders at the facility must be informed that this process will take place in order to facilitate safe implementation of the emergency procedures. After the mitigation process is complete, communication must occur to inform stakeholders that the water is acceptable for general use. The facility must document any emergency mitigation processes that take place.

5. SPECIAL USE WATER SYSTEMS (e.g., HEMODIALYSIS, LABORATORY, PHARMACY COMPOUNDING): It is important to consider the implications of *Legionella* mitigation strategies on special use water systems within the building. For example, chemical disinfectants may result in the introduction of products into, or the formation of disinfection **August 13, 2014 VHA DIRECTIVE 1061 APPENDIX A A-7** byproducts in, the building water supply at concentrations that may be toxic to patients on hemodialysis. Accordingly, the impact of mitigation strategies must account for potential toxicity, methods for removal of the chemical agent and byproducts from the special use water system, and availability of assay methods to measure the chemical agent and byproducts for assuring patient safety. Employees responsible for the oversight of special use water systems are to be consulted during the development and implementation of water treatment strategies for *Legionella* and promptly notified of any changes in treatment procedure.**August 13, 2014 VHA**

APPENDIX B
POLICY AND GUIDELINES FOR MINIMIZING THE RISK OF SCALD INJURY FROM EXPOSURE TO HOT WATER FROM THE POTABLE HOT WATER DISTRIBUTION SYSTEM

1. INSTALLATION OF ANTI-SCALD DEVICES AND MIXING VALVES: One of the primary methods for preventing *Legionella* growth in building potable water distribution systems is water temperature. However, it is not possible to maintain water temperatures at the outlet that will kill *Legionella* bacteria and simultaneously eliminate the possibility of scald injury in persons partially or fully insensitive to hot water temperature, or having delayed or impaired response capabilities.

© 2018 Gamage SD et al. *JAMA Network Open*.

a. **Policy.** In order to allow for hot water temperatures at a level that inhibits *Legionella* growth, buildings subject to this Directive must install mixing valves (can be supplemented with anti-scald devices) at all outlets where end users access water (e.g., sink taps, showers) to regulate the temperature of water at the outlet to prevent scalding. ***NOTE:*** *Mixing valves are to be positioned as close to the outlets as possible, thus allowing for hot water to circulate throughout most of the building potable hot water distribution system(s) at a temperature that will kill Legionella or inhibit growth.*

b. **Water Temperature.** Mixing valves are to regulate water temperature so that water is discharged from outlets at 110 degrees Fahrenheit (°F) or below. ***NOTE:*** *Water temperature for emergency showers and eyewashes must be between 60°F and 100°F.*

c. **Selection of Temperature Regulating Devices.** The medical facility is to determine the type(s) of mixing valve and/or anti-scald guard that will be installed in accordance with VA Plumbing Design Manual/Master Construction Specifications and the International Plumbing Code. ***NOTE:*** *Use of mixing valves could maintain the water temperature between the mixing valve and the end use point (outlet) at a temperature conducive to Legionella growth. Maintaining biocide residual levels at these sites (e.g. with an installed system and/or with periodic flushing) can inhibit Legionella growth. Results from environmental (validation) testing for Legionella can identify areas that may need remedial attention.*

d. **Inspections.** Anti-scald devices and mixing valves must be tested and serviced for proper functioning at least annually in accordance with manufacturers' instructions. More frequent inspections and maintenance of devices and valves may be required, based on the quality of water. Inspections and any performed maintenance must be documented.

2. EQUIPMENT WHERE PATIENTS ARE EXPOSED TO HEATED WATER VIA FULL OR PARTIAL IMMERSION: For equipment where full or partial immersion is the means of patient contact with heated water (e.g., bathtubs, whirlpool tubs, and foot baths), the following measures are required for the prevention of scald injury:

a. Mixing valves at the outlet that are capable of blending the hot and cold water supply to hold water temperatures at or below 110°F are required. ***NOTE:*** *Maximum temperature of water outflow may be reduced based on a risk assessment of users or setting (e.g., Spinal Cord Injury/Disorder Center, Community Living Center).*

b. All patient immersion baths must be equipped with a large digital readout device displaying the bath water temperature. Bath water must not exceed 110°F at the time of patient immersion. The readout temperature must be verified by taking the temperature of the water with a hand held thermometer (preferably non-mercury containing) and comparing this reading with the reading of the tub thermometer. For tubs with an elevated reservoir tank, a remote temperature-sensing probe that can be submerged into the tank water may be utilized to provide the verification temperature. Thermometers and probes must be calibrated, used, and validated in accordance with manufacturers'

© 2018 Gamage SD et al. *JAMA Network Open.*

instructions. ***NOTE:*** *Using sensation alone (e.g., hand, wrist, elbow) is not an acceptable practice for determining safe water temperature*. The actual temperature of water in the tub must be accurately monitored before and during each bath. Consideration needs to be given to the documentation of these temperatures. Facilities must determine an acceptable range of temperature for patient immersion baths not exceeding the maximum limit of 110°F.

3. THERMAL ERADICATION: Thermal eradication (i.e. the raising of the hot water temperature to 160°F - 170°F and the flushing of outlets) is an option for emergency remediation of *Legionella* in hot water distribution systems. If the facility prefers to have the thermal eradication option available, then selection of anti-scald devices and mixing valve that are amenable to this option is recommended. Alternatively, a facility could use a different *Legionella* remediation option, such as shock chlorination on its own or in conjunction with thermal eradication (to the extent feasible with mixing valves present).

APPENDIX C
CLINICAL AND ENVIRONMENTAL VALIDATION OF ENGINEERING CONTROLS FOR PREVENTION OF *LEGIONELLA* GROWTH

1. VALIDATION POLICY: Validation focuses on collecting and evaluating information to determine if the engineering control measures (e.g., water temperature, biocide levels) are effectively controlling *Legionella* growth in a building's potable water distribution system(s). The two required validation methods for VHA buildings subject to this Directive are clinical surveillance testing and environmental water testing. Validation procedures are to be included in the building healthcare-associated (HCA) *Legionella* disease (LD) Prevention Plan. Requirements for the two methods are described below, along with guidance on interpretation of results and remedial actions. Summary flow charts depicting the key concepts for environmental validation and clinical validation are included at the end of this Appendix. ***NOTE:*** *While environmental water testing is listed first in this Appendix for logistical reasons, it is not meant to imply priority or importance over clinical surveillance for disease. Furthermore, both the clinical and environmental validation processes in this Appendix are intended to assess the effectiveness of the routine implementation of engineering controls, not practices that may be necessary after a case of HCA LD or in outbreak settings.*

2. ENVIRONMENTAL VALIDATION: For this policy, environmental validation is the process of testing the building's potable water distribution system(s) to determine if the engineering controls are successfully inhibiting growth of *Legionella*. This section provides overarching requirements and recommendations for water testing and describes mitigation actions. ***NOTE:*** *More detailed requirements and instructions (e.g., sample site selection, sampling procedures) will be provided by the VHA Water Safety Program. See paragraph 5 of this Appendix for a summary flow chart for interpreting routine water testing results.*

© 2018 Gamage SD et al. *JAMA Network Open*.

a. Testing of the building's hot and cold water distribution system(s) for *L. pneumophila* must be performed at least quarterly (once per Federal Fiscal Year quarter).

(1) Water samples from at least 10 outlets on the hot water distribution system and at least 10 outlets on the cold water distribution system must be tested from each building for each quarterly testing cycle. At the time each water sample is taken, test and document the following for each sample: water temperature, level of residual biocide, and pH. ***NOTE:*** *"Outlets" are the points in the water distribution system where the end user comes in contact with the water (e.g., faucets and showers). Facilities may choose to conduct testing of additional outlets or areas (e.g., water tanks, distribution piping). This additional testing should be determined based on local conditions (e.g., size of building, patient population risk factors, known history of HCA LD, suspicion for recent or current HCA LD occurrence, previous environmental testing results, and/or ability to implement engineering controls to prevent Legionella growth in the building water distribution systems). Facilities may choose to take cold and hot water samples from the same outlets; in this case, each sample from the outlet must be collected separately.*

(2) For routine water testing, collection of swab samples is not required, although a facility may choose to collect swab samples in addition to water samples. Swab samples alone do not
meet the requirement of this Directive. ***NOTE:*** *Swab samples, along with water samples, should be collected during a case investigation for paired evaluation.*

b. Once collected, samples are to be processed by a testing laboratory with experience in microbial testing of potable water. The Facility Water Safety Committee is to select the testing laboratory using the following criteria:

(1) Laboratories that process the water samples for *Legionella* must be certified by the Centers for Disease Control and Prevention (CDC) Environmental *Legionella* Isolation Techniques Evaluation (ELITE) program or the Public Health England (PHE) *Legionella* External Quality Assessment (EQA) scheme as proficient at performing the culture of *Legionella* from environmental samples. Information about CDC ELITE certified laboratories can be found at https://wwwn.cdc.gov/elite/Public/MemberList.aspx. Laboratories must also be able to determine if the *Legionella* detected in environmental samples is the species *Legionella pneumophila* serogroup 1. ***NOTE:*** *Per CDC guidance, rapid testing methods, such as polymerase chain reaction (PCR) and direct fluorescent antibody (DFA), are not recommended for the detection of Legionella in environmental water samples.* In addition to the requirement for current CDC ELITE or PHE *Legionella* EQA certification, consider using a laboratory that also has environmental microbiology accreditation by a recognized accrediting program.

(2) Consider selection of a laboratory capable of concentrating water samples prior to plating the samples on selective media to increase the sensitivity of the assay.

© 2018 Gamage SD et al. *JAMA Network Open.*

(3) If there is a possibility that the facility will use molecular typing to characterize environmental *Legionella* isolates, the facility should make arrangements with the testing laboratory for storage of the environmental isolate(s) at least temporarily.

(4) Prior to environmental testing, the selected laboratory should be consulted regarding requirements and recommendations on sample collection and shipping. ***NOTE:*** *The selected laboratory needs to be informed if biocides (e.g. chlorine) have been added to the building's potable water distribution systems to determine if samples need to be processed to neutralize the biocide prior to being sent out for testing.* c. If environmental testing detects *L. pneumophila* then the environmental actions in this sub-paragraph are required. Any amount of *L. pneumophila* detected in a sample is considered a positive result. ***NOTE:*** *For subsequent clinical actions related to Legionella-positive environmental samples, see paragraph 3 of this Appendix.*

(1) Assess the implementation of the engineering controls (e.g., water temperature, biocide levels, etc.) to determine if corrective adjustments need to be taken.

(2) Implement remedial action using the criteria below. This remedial action approach uses a "graded response" for addressing *L. pneumophila*-positive samples detected through routine water testing. That is, while each positive sample will require further assessment, the extent of remediation is situation-dependent. ***NOTE:*** *Consultation with the VHA Water Safety Program is available for guidance on remediation.*

(a) If one outlet is positive for *L. pneumophila*, then a determination must be made as to which other outlets in the area (on same water distribution loop) must be tested for *L. pneumophila* and if any mitigation of the immediate water distribution loop or area is necessary pending the results of any additional cultures. The fixture that tested positive for *L. pneumophila* must be promptly remediated.

(b) If more than one outlet is positive for *L. pneumophila*, assess the results to determine the subsequent actions as follows:

1. If the outlets that tested positive are in the same area of the building or on the same water distribution loop, then promptly conduct remediation of the area or loop.

2. If the outlets that tested positive are in different areas of the building or on different water distribution loops, then promptly conduct remediation in a graded response based on location of the positive outlets. The Facility Water Safety Committee should meet to review the location of the positive water samples in relation to the configurations of the building water distribution system(s) to determine the extent of remediation (i.e., areas/loops versus entire building). For example, if there are two positive sample results in different parts of the building and all other samples are negative, then the facility may determine that remediation of those areas or loops with positive samples is appropriate. If a number of outlets are positive throughout the building, then the facility should strongly consider emergency remediation of the entire water distribution system(s) in the building as described in Appendix A, paragraph 4. Detection of *L. pneumophila* in more than one

© 2018 Gamage SD et al. *JAMA Network Open*.

outlet in different areas of the building water system on repeated cycles of routine quarterly water testing is also a strong indication for remediating the whole system. Facilities should consider local factors (e.g., previous environmental testing results, facility size and configuration of water distribution systems, population risk factors, past history of HCA LD) when determining the extent of remediation to be conducted. ***NOTE:*** *Consultation with the VHA Water Safety Program is available for guidance on positive sample results and remediation efforts.*

3. After environmental remediation is completed, promptly retest the water in the areas that tested positive for *L. pneumophila* to determine if the remediation procedures were successful at reducing *L. pneumophila* to undetectable levels. If the remediation procedures were successful, then the quarterly water environmental validation cycle is complete. If the remediation procedures did not reduce *L. pneumophila* to undetectable levels, assess the post-remediation results for location of positive samples, and determine subsequent actions based on the guidance in this section. ***NOTE****: This re-testing of the water distribution system(s) for Legionella as post-remediation follow-up is not to be considered as the next round of routine quarterly environmental testing.*

4. Documentation is required for environmental testing (date, outlets, and results), any assessments of positive results, any remedial action taken, and efficacy of remedial actions. A report of these items is to be submitted to the Facility Water Safety Committee, Safety Committee, and Infection Control Committee.

d. If environmental water testing detects *Legionella* but the *Legionella* is not *L. pneumophila*, then the following actions are required since growth of non-*pneumophila Legionella* species indicates that conditions are conducive for growth of *L. pneumophila*:

(1) Assess the engineering controls (e.g., water temperature, biocide levels) and flushing protocols and make adjustments, as necessary, for *Legionella* control.

(2) Further testing for *L. pneumophila* of the water distribution system(s) is required to determine if *L. pneumophila* is present. ***NOTE:*** *It is advisable to focus this testing on at least areas where positive water samples were identified.*

(a) If *L. pneumophila* is found, then follow the procedures outlined above in this Appendix in paragraph 2.c.

(b) If *L. pneumophila* is not found, then the quarterly environmental validation cycle is complete. ***NOTE:*** *Facilities may choose to follow some or all of the activities outlined above in paragraph 2.c. even if only non-*pneumophila *Legionella species are detected if the Facility Water Safety Committee determines such actions are warranted (e.g. if the facility has a history of HCA LD caused by a non-*L. pneumophila *strain, if* L. pneumophila *has been detected in the facility water distribution previously, or if the building includes areas with persons at a particularly heightened risk for development of infectious diseases such as a transplant unit or hematology-oncology unit).*

3. CLINICAL VALIDATION: Clinical validation is the process of determining if the primary engineering controls and any supplemental water treatment systems are successfully inhibiting *Legionella* growth in the potable water distribution system(s) by monitoring the occurrence of HCA LD. ***NOTE:*** *This Directive focuses on prevention of HCA LD. If a case of HCA LD is detected, this Directive provides policy and guidance for actions to prevent further cases. Policy and guidance for full case investigations of confirmed or suspected HCA LD is not encompassed within the scope of this Directive. Further guidance on this topic will be provided by the VHA Water Safety Program; the VHA Water Safety Program is also available for consultative assistance. See paragraph 6 of this Appendix for a summary flow chart for Clinical Validation.*

a. **Clinical testing.** Consider diagnostic *Legionella* testing for patients who have pneumonia (community- or healthcare-associated); such testing is to be conducted if clinically indicated. Diagnosis of LD relies principally on use of *Legionella* urinary antigen testing which only detects disease caused by *L. pneumophila* serogroup 1. Culture of sputum or other respiratory specimens for *Legionella* is also to be considered if these types of clinical specimens are collected during the course of clinical management of the case. If there are known cases of LD in the community (e.g., at other healthcare facilities in the surrounding area, from a known higher incidence of LD in the geographic area, or alerts from the Health Department) then it would be prudent to maintain a high index of suspicion for LD for patients that present to the facility with pneumonia. If a case of LD is diagnosed, see paragraph 3.c. of this Appendix for determination of linkage of the case to the healthcare facility.

b. **Circumstances for Heightened Awareness for Clinical Testing.** If *L. pneumophila* is found during environmental validation testing then clinicians are to be vigilant for patients who develop respiratory disease or processes suggestive of pneumonia (e.g., fever with cough or production of sputum, or radiographic findings) during their hospital admission or while under residential care and perform testing of such identified patients for LD. Also consider heightened awareness for clinical testing if a case of definite or possible HCA LD is detected for the building or if there is a community-based outbreak of LD, ***NOTE:*** *For the situation in which environmental testing indicates positive results, the results must be communicated to clinical providers in order to have heightened awareness for clinical testing (see paragraph 4 of this Appendix). If the Legionella detected during environmental water sampling is* L. pneumophila *serogroup 1, then use of both urinary antigen testing and sputum culture for diagnosis of LD is appropriate. If Legionella other than* L. pneumophila *serogroup 1 is found during the environmental water sampling, then use of sputum culture for diagnosis of LD is appropriate; it is also reasonable to consider urinary antigen testing in addition to culture testing since* L. pneumophila *serogroup 1 may also be present in the water distribution system.*

(1) This heightened state of clinical vigilance and testing as a result of positive environmental samples is to be followed for at least 3 months and until the next round of quarterly water testing results are received. If *L. pneumophila* is again found in the building's water distribution system at the next quarterly testing, then the facility is to remediate the water distribution system and the heightened state of clinical vigilance

© 2018 Gamage SD et al. *JAMA Network Open.*

continues for another 3 months. If *L. pneumophila* is not found in the building's water system, then the state of heightened clinical vigilance can end and the facility can return to conducting routine clinical testing.

(2) If a case of LD is diagnosed at the facility, see paragraph 3.c. of this Appendix for determination of linkage of the case to the healthcare facility.

c. **Determination of Epidemiological Linkage of LD Cases to the Healthcare Facility for Assessment of Remediation Needs.** If diagnostic testing determines that any case of pneumonia is caused by any species of *Legionella*, then determine if the case is epidemiologically-linked to the building using the following guidance:

(1) If the person has spent 10 or more days continuously in a VHA facility prior to the onset of pneumonia symptoms, then the case is classified as **definite HCA LD**. The remedial actions in paragraph 3.d. below are required.

(2) Based on CDC 2003 written guidelines for prevention of healthcare-associated pneumonia, if the person has spent between 2 and 9 days in a VHA facility prior to the onset of pneumonia symptoms, then, for the purposes of this document, the case is classified as **possible HCA LD (inpatient)**. Recently, the CDC updated its surveillance Legionellosis Case Report Form (January 2014) in which a possible HCA LD case is classified if the patient had exposure to a healthcare facility for a portion of the 10 days prior to onset of illness, regardless of whether that contact occurred during an inpatient stay or outpatient visit; for the purposes of this document, this is considered a **possible HCA LD (inpatient and outpatient)**. The facility needs to assess possible association of a LD case with the facility on a case by case basis. Conduct the following actions to determine if remediation of the facility water system(s) is necessary:

(a) Collect environmental water and swab samples from the hot and cold water distribution loops in at least the areas where the patient had contact with the building water distribution systems. ***NOTE:*** *Consider taking additional water and swab samples from areas where the patient may have been exposed to water, such as ice machines, medical devices and equipment. Also consider non-domestic water sources, such as outdoor fountains and cooling towers, if epidemiologically indicated.* The environmental samples are to be processed for the culture of *Legionella* bacteria to determine if the species responsible for the person's illness is detected in environmental samples.

(b) If any of the environmental testing results are positive for *L. pneumophila*, or the species of *Legionella* associated with the case if the case was not caused by *L. pneumophila*, then the remedial actions in paragraph 3.d. below are required.

(c) If the environmental testing does not detect *L. pneumophila*, or the *Legionella* species associated with the case, in the environmental samples, then clinical staff are to remain vigilant for the identification of additional cases of LD for at least the next 3 months. Any LD-positive diagnostic result precipitates the actions in paragraph 3.c. above to determine if the case is linked to the healthcare facility. ***NOTE:*** *Based on local conditions or*

circumstances, the Facility Water Safety Committee may determine that remediation is to be conducted even if the criteria above are not met. Consultation with the VHA Water Safety Program is available for guidance.

(3) If the facility compares patient and environmental isolates by molecular typing and the isolates are determined to be a match, then the case is epidemiologically-linked to the facility regardless of the duration of time the patient spent in the facility or the nature of the visit. The remedial actions in paragraph 3.d. below are required.

(4) If a HCA LD case is identified (whether definite, possible, or molecularly-linked), then implement heightened awareness for clinical testing as described in paragraph 3.b. of this Appendix for a period of at least 3 months. d. **Remedial actions.** Linkage of a case of LD to the facility as described in paragraph 3.c. above requires the following prompt actions:

(1) The Facility Water Safety Committee must meet and review data on the implementation of the primary engineering controls and any supplemental water treatment systems to determine if there were any circumstances (e.g., construction activities, reduced water temperatures, reduced biocide residual levels) that could have resulted in *Legionella* growth and that require corrective action to restore the controls and systems to levels for ongoing suppression of *Legionella* growth. ***NOTE:*** *If the Facility Water Safety Committee discusses any case of LD, patient information must be de-identified prior to sharing the information with the committee.*

(2) Implement emergency remediation, described in Appendix A, paragraph 4, as an immediate remediation of the potable water distribution system(s) in the building. Consider cleaning ice machines, medical devices and/or equipment as a way to prevent potential further transmission of *Legionella* if such apparatus may be associated with the diagnosed case. In the situation of a definite HCA LD case where taking environmental samples may not have been necessary to link the case to the facility, it may be prudent to collect environmental samples prior to implementing remediation to determine the extent of *Legionella* in the building water distribution system(s); however, remediation is not to be delayed until results are returned and not to be canceled if results are all negative. Promptly retest the water after remediation is complete to determine if the remediation was successful at reducing *Legionella* to undetectable levels; detection of *Legionella* in subsequent environmental testing prompts a review of remediation procedures and further remediation and testing to reduce *Legionella* to undetectable levels. ***NOTE:*** *Consultation with the VHA Water Safety Program is available for guidance on remediation.*

4. COMMUNICATION OF VALIDATION ACTIVITIES AND RESULTS: *Legionella* prevention and control in the healthcare setting is multidisciplinary; effective prevention of HCA LD requires timely communication among staff. This is especially important regarding interpretation and follow-up for positive validation results as delineated in this Appendix since positive results in one section trigger further actions, some of which may affect actions required in other sections.

a. If the results of the environmental validation are positive for *L. pneumophila*, the following must be promptly notified: Chief of Staff, the Associate Director for Patient Care Services, the Facility Water Safety Committee, Infection Prevention and Control, and Facility Chief Engineer or Facility Manager. The Chief of Staff and the Associate Director for Patient Care Services are responsible for ensuring that clinicians involved in direct patient care are notified of the positive environmental results, including the species and serogroup detected (if appropriate), in order to implement heightened awareness for clinical testing for LD as required in paragraph 3.b of this Appendix, and in accordance with the building HCA LD prevention plan.

b. If there is a definite or possible HCA LD case at the facility, Infection Prevention and Control is to promptly notify the Chief of Staff, the Associate Director for Patient Care Services, and the Facility Water Safety Committee. This notification of the Facility Water Safety Committee will initiate their review of engineering controls and implementation of the emergency remediation process as described above in paragraph 2.c.

© 2018 Gamage SD et al. *JAMA Network Open*.

5. SUMMARY FLOW CHART FOR INTERPRETATION OF ROUTINE QUARTERLY ENVIRONMENTAL WATER TESTING RESULTS IN THE ABSENCE OF HCA LD: This flow chart summarizes the main concepts in Appendix C regarding Environmental Validation processes. For a summary of Clinical Validation processes, see the flow chart in paragraph 6. For details on these validation processes, see the text in Appendix C.

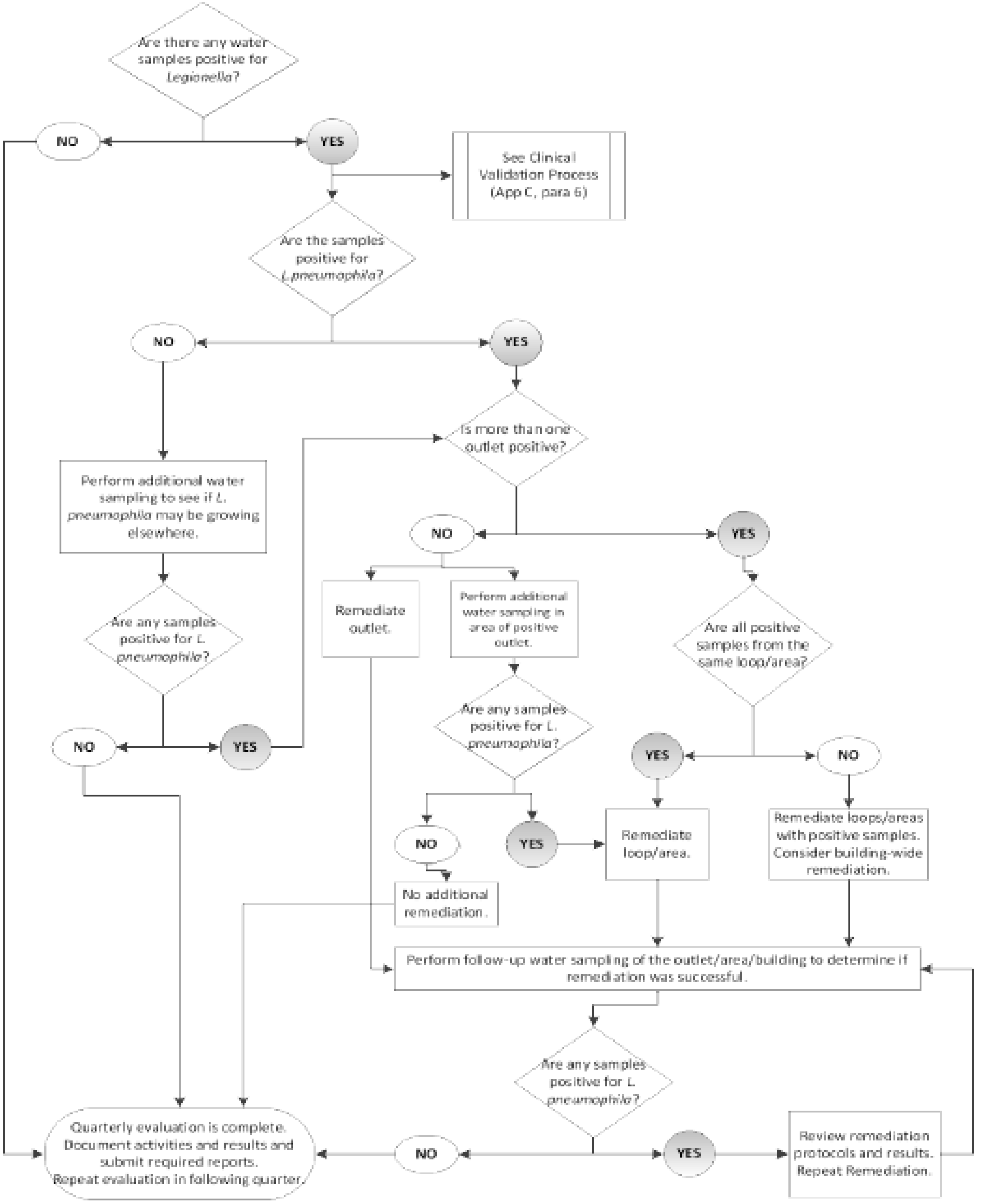

© 2018 Gamage SD et al. *JAMA Network Open.*

6. SUMMARY FLOW CHART FOR CLINICAL VALIDATION: This flow chart summarizes the main concepts in Appendix C regarding Clinical Validation processes. For a summary of Environmental Validation processes, see the flow chart in paragraph 5. For details on these validation processes, see the text in Appendix C.

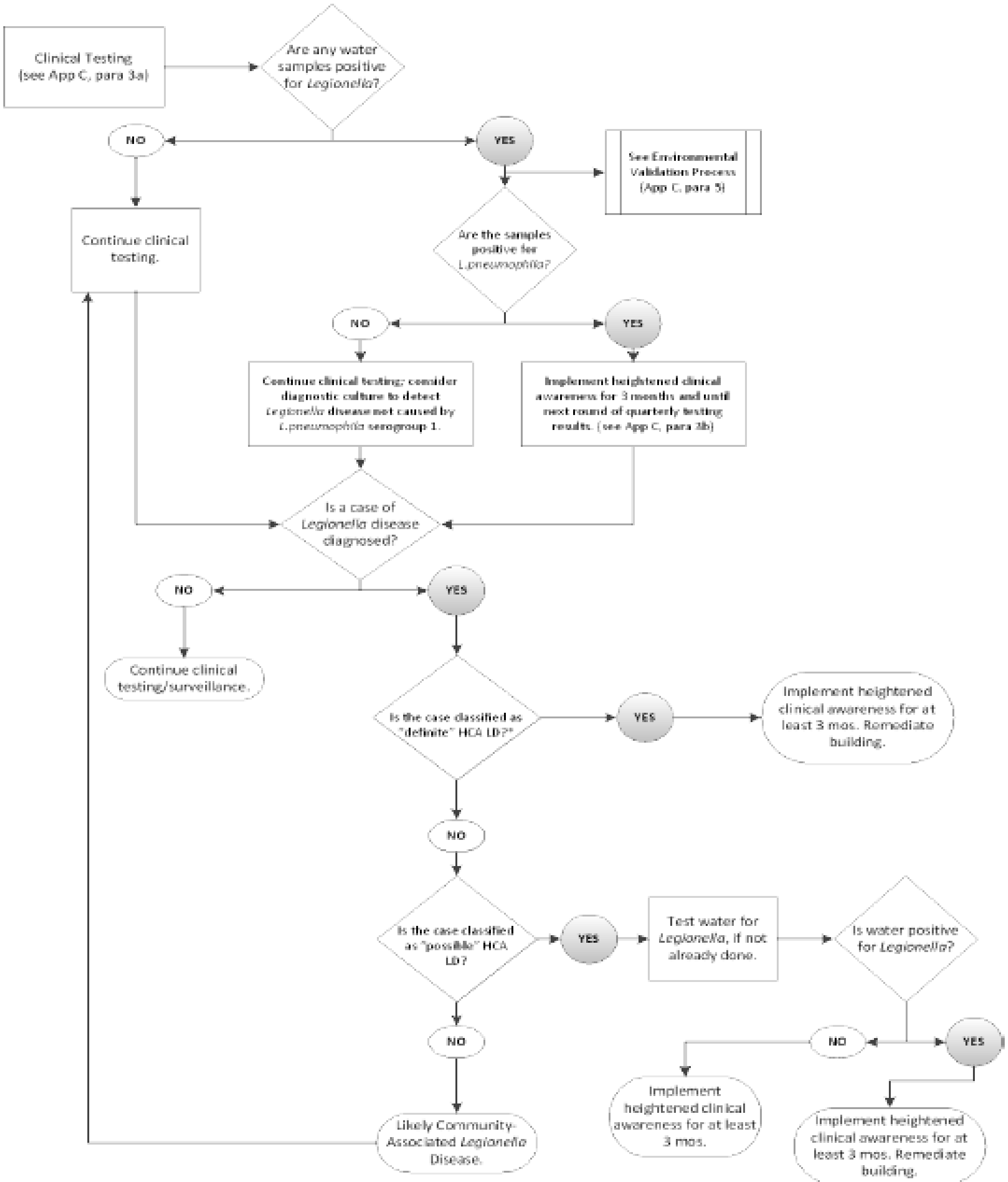

* If molecular typing was done and the patient *Legionella* isolate matches an environmental *Legionella* isolate, then follow the "Yes" arm of this decision box to trigger the actions associated with a definite case of HCA LD.

© 2018 Gamage SD et al. *JAMA Network Open.*
